# Synthesis and Anticancer Activities of Glycyrrhetinic Acid Derivatives

**DOI:** 10.3390/molecules21020199

**Published:** 2016-02-06

**Authors:** Yang Li, Ling Feng, Zhi-Fang Song, Hai-Bei Li, Qi-Yong Huai

**Affiliations:** 1 Marine College, Shandong University, Weihai 264209, China; lychemlh@163.com (Y.L.); fenglingjpf@163.com (L.F.); zhifangdl@163.com (Z.-F.S.); 2 Zhong Yuan Academy of Biological Medicine, Liaocheng People’s Hospital/Affiliated Liaocheng Hospital, Taishan Medical University, Liaocheng 252000, China

**Keywords:** glycyrrhetinic acid (GA), derivatives, anticancer, MCF-7, MDA-MB-231

## Abstract

A total of forty novel glycyrrhetinic acid (GA) derivatives were designed and synthesized. The cytotoxic activity of the novel compounds was tested against two human breast cancer cell lines (MCF-7, MDA-MB-231) *in vitro* by the MTT method. The evaluation results revealed that, in comparison with GA, compound **42** shows the most promising anticancer activity (IC_50_ 1.88 ± 0.20 and 1.37 ± 0.18 μM for MCF-7 and MDA-MB-231, respectively) and merits further exploration as a new anticancer agent.

## 1. Introduction

Breast cancer is one of the most common diseases amongst women throughout the world. About 521,900 women lost their lives because of it in 2012, 197,600 of which were from developed countries and 324,300 from developing countries [1]. In 2016, 249,260 new breast cancer cases and 40,890 breast cancer deaths are projected to occur in the United States [2]. A current hot research topic is how to develop novel therapeutics with improved selectivity and higher anticancer activity by chemical modifications of natural sources. Up to now, a large number of chemotherapeutic agents derived from natural products and used for the treatment of cancer, have shown satisfactory therapeutic effects, like vinblastine, vincristine, the camptothecin derivatives, e.g., topotecan, irinotecan and etoposide, were derived from epipodophyllotoxin and paclitaxel [3].

Terpenoids are the largest group of natural compounds found in plants. Among terpenoids, a large number of triterpenoids exhibit cytotoxicity against a variety of tumor cells as well as anticancer efficacy in preclinical animal models [4,5,6]. Those triterpenoids regulate tumor cell proliferation, transformation, survival, invasion, angiogenesis, metastasis, chemoresistance and radioresistance [7]. As a kind of triterpenoid, glycyrrhetinic acid (GA) has many valuable pharmacological properties, such as antiviral [8,9], anti-allergic [10], anti-inflammatory [11,12], anti-ulcer [13] and anticancer activity [14,15]. It has been reported that GA exhibited selective toxicity to varieties of tumor cells, making it an ideal lead compound for anticancer treatment [16,17]. Ferulic acid as a natural product that also has apparent biological activities like antibacterial [18], anti-inflammatory [19,20] and anticancer properties [21]. To improve the cytotoxicity of GA, many researchers have tried to enhance its potency by various derivatizations. Some studies have shown that the addition of lipophilic fragments to antitumor molecules could increase their anticancer activity [22,23,24,25]. In addition, natural products which were conjugated with amino acids provide improved bioacitivity [26,27,28]. In this study, based on the pro-drug principle and the previously reported therapeutic potential of GA, we designed and synthesized a series of novel GA derivatives in which the 30-carboxyl group was coupled with lipophilic fragments (ferulic acid analogs) and the 3-hydroxyl group was coupled with amino acids (l-methionine or l-selenomethionine) to improve the anticancer potency of GA.

In totally, forty derivatives of GA were successfully synthesized and and their structures characterized by ^1^H-NMR, ^13^C-NMR, MS and elemental analysis. Their *in vitro* anticancer activities were then tested, using a MTT assay, against two human breast cancer cell lines (MCF-7 and MDA-MB-231) and one normal human retinal pigment epithelial cell line (hTERT-RPE1 cells). Most of the derivatives exhibited much stronger inhibitory activity than GA against those two breast cancer cell lines (but lower than the positive control doxorubicin) and relatively lower inhibitory activity against normal cells. More importantly, one derivative, compound **42** (see Section 2 below), showed significantly stronger cytotoxicity against both MCF-7 cells and MDA-MB-231 cells than GA itself. Our data suggested that coupling lipophilic fragments (especially ferulic acid methyl ester) and amino acids (especially l-selenomethionine) to GA is a promising approach to generate highly active anticancer compounds. Further SAR development is in progress to discover more potential lead antitumor drugs.

## 2. Results and Discussion

### 2.1. Chemistry

**Scheme 1:**
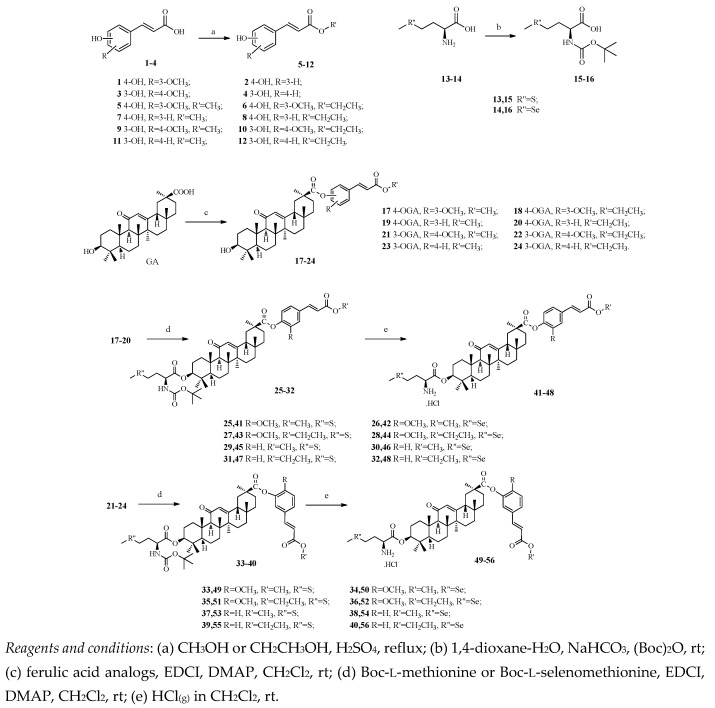
Preparation of glycyrrhetinic acid derivatives **17**–**56**.

The synthesis of new GA derivatives **17**–**56** was carried out according to the steps shown in Scheme 1.

All these derivatives are new compounds which were not previously reported. The lipophilic fragments **5**–**12** were obtained by the treatment of **1**–**4** with methanol or ethanol catalyzed by concentrated sulfuric acid. To avoid the formation of byproducts, we used the *t*-butyloxycarbonyl (Boc-) group to protect amino acids and thus obtained compounds **15**–**16**, which were used in the next step without further purification.

Compounds **17**–**24** were obtained through the formation of an ester bond between compounds **5**–**12** and GA after stirring for 12 h at room temperature in dry dichloromethane (DCM) catalyzed by 1-(3-dimethylaminopropyl)-3-ethylcarbodiimide hydrochloride (EDCI) and 4-dimethylamino-pyridine (DMAP). We applied the same method to introduce the N-Boc group to protect amino acids at position C-3 and obtained compounds **25**–**40**. To get compounds **41**–**56**, deprotection was performed by treating the compounds with dry HCl gas in DCM.

### 2.2. Anticancer Activities

The anticancer activities of compounds **17**–**24**, **41**–**56** against MCF-7, MDA-MB-231 and hTERT-RPE1 cells were determined *in vitro* by an MTT assay. Doxorubicin was included in the experiments as positive control, and the IC_50_ of GA is also presented to compare the anticancer activities. The data was calculated and presented as IC_50_ values in Table 1. All the derivatives **17**–**24**, **41**–**56** showed significant cytotoxicities, in which their IC_50_ values ranged from 1.88 ± 0.20 to 24.45 ± 1.36 μM on MCF-7 cells and from 1.37 ± 0.18 to 23.15 ± 1.07 μM on MDA-MB-231 cells, while the IC_50_ values of the parent compound GA are 75.66 ± 1.52 and 84.70 ± 1.73 μM, respectively. Among the derivatives, compound **42** showed the highest cytotoxicity with the lowest IC_50_ values of 1.88 ± 0.20 μM on MCF-7 cells and 1.37 ± 0.18 μM on MDA-MB-231 cells, which were 40.24 and 61.82 times better than GA.

**Table 1:** IC_50_ values (in μM) of test compounds against MCF-7, MDA-MB-231 and hTERT-RPE1 cell lines.

Compd.	IC_50_(μM) ^a^			Compd.	IC_50_(μM) ^a^		
	MCF-7	MDA-MB-231	hTERT-RPE1		MCF-7	MDA-MB-231	hTERT-RPE1
**GA**	75.66± 1.52	84.70± 1.73	63.41 ± 1.07	**45**	7.24 ± 0.30	6.43 ± 0.84	8.48 ± 0.73
**17**	13.64±0.93	5.03 ± 0.82	17.32 ± 1.21	**46**	6.02 ± 0.35	6.27 ± 0.24	6.33 ± 0.19
**18**	22.46 ± 1.26	8.14 ± 0.76	22.80 ± 0.97	**47**	2.65 ± 0.12	2.31 ± 0.65	5.65 ± 1.02
**19**	20.29 ± 1.47	14.38 ± 0.52	29.63 ± 1.16	**48**	2.42 ± 0.23	1.86 ± 0.29	7.08 ± 0.73
**20**	24.45 ± 1.36	14.46 ± 0.58	28.41 ± 0.87	**49**	8.70 ± 0.14	4.56 ± 0.36	8.92 ± 0.55
**21**	8.54 ± 0.67	7.31 ± 0.16	18.59 ± 0.54	**50**	5.12 ± 0.58	3.13 ± 0.45	9.79 ± 0.37
**22**	19.27 ± 1.01	9.41 ± 1.03	21.11 ± 0.73	**51**	8.81 ± 0.20	4.67 ± 0.74	12.52 ± 0.40
**23**	14.90 ± 0.75	20.84 ± 1.20	24.09 ± 0.88	**52**	6.93± 0.63	3.27 ± 0.66	10.06 ± 0.28
**24**	19.30 ± 0.98	23.15 ± 1.07	22.88 ± 0.68	**53**	7.20 ± 0.55	3.54 ± 0.46	8.49 ± 0.51
**41**	6.00 ± 0.43	3.52 ± 0.61	10.36 ± 0.80	**54**	3.48 ± 0.82	2.46 ± 0.77	4.55 ± 0.44
**42**	1.88 ± 0.20	1.37 ± 0.18	4.93 ± 0.36	**55**	5.79 ± 0.85	1.92 ± 0.91	6.63 ± 0.22
**43**	8.62 ± 0.23	5.36 ± 0.44	16.28 ± 0.51	**56**	2.57 ± 0.40	1.53 ± 0.25	3.70 ± 0.29
**44**	8.45 ± 0.32	3.49 ± 0.61	12.33 ± 0.46	ADR ^b^	0.49 ± 0.09	0.53 ± 0.14	2.76 ± 0.11

All values are given as means ± standard deviation. ^a^ IC_50_ is the drug concentration effective in inhibiting 50% of the cell growth measured by MTT method. ^b^ The drug doxorubicin (ADR) was used as positive control in this study.

In addition, it was observed that the introduction of different lipophilic fragments on the 30-carboxyl group or different amino acids on the 3-hydroxy group of GA results indifferent cytotoxicities. When we combined ferulic acid methyl ester analogs at C-30, the cytotoxicity was higher than with the corresponding ethyl ester analogs (the IC_50_ values: **17** < **18**, **19** < **20**, **21** < **22**, **23** < **24**). It is also found that the lipophilic fragments with a methoxy group have higher anticancer activity (the IC_50_ values: **17** < **19**, **18** < **20**, **21** < **23**, **22** < **24**). Meanwhile the introduction of l-selenomethionine at position C-3 (compounds **42**, **44**, **46**, **48**, **50**, **52**, **54**, and **56**), leads to lower IC_50_ values than l-methionine (compounds **41**, **43**, **45**, **47**, **49**, **51**, **53**, and **55**). Almost all the new synthetic GA derivatives exhibited much stronger inhibitory activity against MDA-MB-231 cells and relatively lower inhibitory activity against MCF-7 cells than GA itself.

## 3. Experimental Section

### 3.1. General Information

All the chemicals and reagents were commercially available and used without further purification. Routine thin-layer chromatography (TLC) was performed on silica gel plates (silica gel GF254 from Qingdao Haiyang Chemical Co., Ltd., Qingdao, China), and visualization was performed using UV. ^1^H-NMR and ^13^C-NMR spectra were recorded on a Bruker-400 instrument (Bruker, Billerica, MA, USA) at room temperature with TMS as an internal standard and CDCl_3_ or DMSO-*d*_6_ as solvents. Chemical shifts are expressed in δ (ppm) and coupling constants (*J*) in Hz. Mass spectra were recorded with a MSQ Plus mass spectrometer (Thermo Scientific, Waltham, MA, USA). Melting points were measured by an SGWX-4 micro melting point apparatus (Shanghai Precision & Scientific Instrument Co. Ltd., Shanghai, China) and are uncorrected.

### 3.2. General Method for Synthesizing Compounds **5**–**12**

Ferulic acid (**1**) or *trans*-4-hydroxycinnamic acid (**2**) or isoferulic acid (**3**) or *trans*-3-hydroxycinnamic acid (**4**) and five drops of H_2_SO_4_ (95%) were refluxed in methanol or ethanol for 12 h. The reaction mixture was concentrated *in vacuo* and the residue was dissolved in ethyl acetate. The organic layer was washed with a 5% aqueous NaHCO_3_ solution and water. After drying over anhydrous Na_2_SO_4_, the ethyl acetate was removed *in vacuo*. The residue was purified by column chromatography on silica gel using ethyl acetate/petroleum ether mixtures as eluents to afford compounds **5**–**12**.

### 3.3. General Method for Synthesizing Compounds **15**–**16**

The appropriate amino acid (**13** or **14**, 1 equiv.) and sodium bicarbonate (3 equiv.) was dissolved in a 1:1 mixture of water and 1,4-dioxane. Di-*tert*-butyl dicarbonate (1.2 equiv.) was added and the mixture was stirred at room temperature for 12 h. The 1,4-dioxane was removed under reduced pressure and the mixture was extracted with ethyl acetate. Then the solution was acidified using 1 M hydrochloric acid solution and extracted three times with ethyl acetate. The combined organic layers were washed with brine, dried over sodium sulfate, filtered, and the solvent was evaporated. The crude protected amino acids **15**–**16** were used without any further purification.

### 3.4. General Method for Synthesizing Compouds **17**–**24**

GA (485 mg, 1 mmol) was dissolved in dry DCM (30 mL) and stirred at room temperature for 5 min. Then EDCI (230 mg, 1.2 mmol), DMAP (24 mg, 0.2 mmol) and compounds **5**–**12** (1 mmol) were added to the solution, and then the reaction mixture was stirred at room temperature for 12 h. The organic layer was washed with 1 M HCl solution and concentrated *in vacuo*. The residue was purified by column chromatography on silica gel with ethyl acetate/petroleum as eluent to yield pure compounds **17**–**24**.

*Ferulic acid methyl ester 3β-hydroxy-11-oxo-olean-12-en-30-oate* (**17**). Compound **17** was obtained from GA and ferulic acid methyl ester as a white solid (548.5 mg, 83%); m.p. 229–231 °C; ^1^H-NMR (CDCl_3_): δ 0.72–0.74 (m, 1H, H-5), 0.83 (s, 3H, H-28), 0.90 (s, 3H, H-24), 0.96–1.00 (m, 1H, H-1’), 1.03 (s, 3H, H-23), 1.08–1.11 (m, 1H, H-15’), 1.16 (s, 3H, H-26), 1.17 (s, 3H, H-25), 1.23–1.25 (m, 1H, H-16’), 1.27–1.30 (m, 2H, H-22’ and 21’), 1.37 (s, 3H, H-29), 1.42 (s, 3H, H-27), 1.44–1.45 (m, 1H, H-22), 1.46–1.48 (m, 1H, H-7’), 1.49–1.51 (m, 1H, H-6’), 1.61–1.63 (m, 1H, H-6), 1.65 (dd, *J* = 13.2, 4.0 Hz, 1H, H-19’), 1.67–1.68 (m, 1H, H-2’), 1.70–1.73 (m, 1H, H-7), 1.77–1.79 (m, 1H, H-2), 1.89 (ddd, *J* = 14.4, 14.4, 5.2 Hz, 1H, H-16), 2.02–2.04 (m, 1H, H-21), 2.08–2.10 (m, 1H, H-15), 2.12 (dd, *J* = 13.2, 4.0 Hz, 1H, H-18), 2.37 (s, 1H, H-9), 2.43–2.45 (m, 1H, H-19), 2.80 (ddd, *J* = 13.2, 3.2, 3.2 Hz, 1H, H-1), 3.25 (dd, *J* = 10.8, 5.6 Hz, 1H, H-3), 3.84 (s, 3H, COOCH_3_), 3.89 (s, 3H, OCH_3_), 5.73 (s, 1H, H-12), 6.42 (d, *J_trans_* = 12.8 Hz, 1H, H-β), 7.02 (d, *J* = 6.8 Hz, 1H, Bn-H-5), 7.13 (s, 1H, Bn-H-3), 7.15 (d, *J* = 6.8 Hz, 1H, Bn-H-6), 7.68 (d, *J_trans_* = 12.8 Hz, 1H, H-α); ^13^C-NMR (CDCl_3_): δ 200.10, 174.43, 168.44, 166.88, 151.40, 149.95, 147.92, 128.43, 123.12, 121.17, 118.34, 113.48, 109.11, 78.66, 61.63, 55.68, 54.84, 51.64, 48.00, 45.33, 44.37, 43.11, 41.21, 38.04, 38.02, 37.42, 37.06, 32.75, 31.81, 31.13, 28.47, 28.05, 28.04, 27.28, 26.40, 26.35, 23.38, 18.59, 17.44, 16.30, 15.57; ESI-MS: *m*/*z* = 661.26 [M + H]^+^. Anal. Calcd. for C_41_H_56_O_7_ (660.40): C, 74.51; H, 8.54%. Found: C, 74.48; H, 8.56%.

*Ferulic acid ethyl ester 3β-hydroxy-11-oxo-olean-12-en-30-oate* (**18**). Compound **18** was obtained from GA and ferulic acid ethyl ester as a white solid (573.7 mg, 85%); m.p. 224–226 °C; ^1^H-NMR (CDCl_3_): δ 0.72–0.74 (m, 1H, H-5), 0.83 (s, 3H, H-28), 0.90 (s, 3H, H-24), 0.96–1.00 (m, 1H, H-1’), 1.03 (s, 3H, H-23), 1.08–1.11 (m, 1H, H-15’), 1.16 (s, 3H, H-26), 1.17 (s, 3H, H-25), 1.23–1.25 (m, 1H, H-16’), 1.27–1.30 (m, 2H, H-22’ and H-21’), 1.37 (t, *J* = 5.6 Hz, 3H, CH_3_), 1.38 (s, 3H, H-29), 1.42 (s, 3H, H-27), 1.43–1.46 (m, 1H, H-22), 1.47–1.48 (m, 1H, H-7’), 1.49–1.51 (m, 1H, H-6’), 1.62–1.64 (m, 1H, H-6), 1.65 (dd, *J* = 13.2, 4.0 Hz, 1H, H-19’), 1.67–1.68 (m, 1H, H-2’), 1.70–1.72 (m, 1H, H-7), 1.76–1.78 (m, 1H, H-2), 1.89 (ddd, *J* = 14.0, 14.0, 4.8 Hz, 1H, H-16), 2.03–2.05 (m, 1H, H-21), 2.08–2.10 (m, 1H, H-15), 2.12 (dd, *J* = 12.8, 3.6 Hz, 1H, H-18), 2.37 (s, 1H, H-9), 2.43–2.45 (m, 1H, H-19), 2.80 (ddd, *J* = 13.6, 3.6, 3.6 Hz, 1H, H-1), 3.25 (dd, *J* = 11.2, 5.2 Hz, 1H, H-3), 3.88 (s, 3H, OCH_3_), 4.30 (q, *J* = 5.6 Hz, 2H, CH_2_CH_3_), 5.73 (s, 1H, H-12), 6.41 (d, *J_trans_* = 12.8 Hz, 1H, H-β), 7.02 (d, *J* = 6.8 Hz, 1H, Bn-H-5), 7.13 (s, 1H, Bn-H-3), 7.15 (d, *J* = 6.8 Hz, 1H, Bn-H-6), 7.67 (d, *J_trans_* = 12.8 Hz, 1H, H-α); ^13^C-NMR (CDCl_3_): δ 200.02, 174.30, 168.32, 166.80, 151.34, 149.95, 147.90, 128.45, 123.01, 121.12, 118.24, 113.47, 109.10, 78.63, 61.75, 60.63, 55.70, 54.87, 47.98, 45.32, 44.36, 43.13, 41.14, 38.00, 37.94, 37.56, 36.93, 32.65, 31.91, 31.15, 28.48, 28.06, 28.04, 27.25, 26.45, 26.38, 23.33, 18.63, 17.39, 16.32, 15.52, 14.28; ESI-MS: *m*/*z* = 675.23 [M + H]^+^. Anal. Calcd. for C_42_H_58_O_7_ (674.42): C, 74.74; H, 8.66%. Found: C, 74.70; H, 8.69%.

*trans-4-Hydroxycinnamic acid methyl ester 3β-hydroxy-11-oxo-olean-12-en-30-oate* (**19**)*.* Compound **19** was obtained from GA and *trans*-4-hydroxycinnamic acid methyl ester as a white solid (530 mg, 84%); m.p. 202–205 °C; ^1^H-NMR (CDCl_3_): δ 0.69–0.72 (m, 1H, H-5), 0.81 (s, 3H, H-28), 0.86 (s, 3H, H-24), 0.94–0.98 (m, 1H, H-1’), 1.01 (s, 3H, H-23), 1.06–1.09 (m, 1H, H-15’), 1.14 (s, 3H, H-26), 1.14 (s, 3H, H-25), 1.19–1.21 (m, 1H, H-16’), 1.24–1.26 (m, 2H, H-22’ and H-21’), 1.35 (s, 3H, H-29), 1.40 (s, 3H, H-27), 1.43–1.45 (m, 1H, H-22), 1.47–1.48 (m, 1H, H-7’), 1.50–1.53 (m, 1H, H-6’), 1.60–1.62 (m, 1H, H-6), 1.64 (dd, *J* = 13.2, 4.0 Hz, 1H, H-19’), 1.67–1.69 (m, 1H, H-2’), 1.72–1.74 (m, 1H, H-7), 1.75–1.78 (m, 1H, H-2), 1.87 (ddd, *J* = 14.8, 14.8, 5.6 Hz, 1H, H-16), 2.04–2.06 (m, 1H, H-21), 2.11–2.13 (m, 1H, H-15), 2.24 (dd, *J* = 13.6, 4.4 Hz, 1H, H-18), 2.35 (s, 1H, H-9), 2.43–2.46 (m, 1H, H-19), 2.78 (ddd, *J* = 12.8, 2.8, 2.8 Hz, 1H, H-1), 3.23 (dd, *J* = 10.4, 6.0 Hz, 1H, H-3), 3.81 (s, 3H, COOCH_3_), 5.67 (s, 1H, H-12), 6.41 (d, *J_trans_* = 16.0 Hz, 1H, H-β), 7.08 (d, *J* = 8.8 Hz, 2H, Bn-H-2 and 6), 7.55 (d, *J* = 8.8 Hz, 2H, Bn-H-3 and 5), 7.68 (d, *J_trans_* = 16.0 Hz, 1H, H-α); ^13^C-NMR (CDCl_3_): δ 200.03, 174.86, 168.65, 166.93, 162.26, 145.61, 132.27, 129.25, 128.70, 122.12, 118.41, 78.74, 61.82, 54.94, 51.86, 48.43, 45.36, 44.39, 43.21, 41.11, 39.16, 39.09, 37.75, 37.10, 32.69, 31.88, 31.13, 28.54, 28.09, 28.06, 27.32, 26.41, 26.38, 23.49, 18.65, 17.49, 16.47, 15.62; ESI-MS: *m*/*z* = 631.21 [M + H]^+^. Anal. Calcd. for C_40_H_54_O_6_ (630.39): C, 76.16; H, 8.63%. Found: C, 76.13; H, 8.67%.

*trans-4-Hydroxycinnamic acid ethyl ester 3β-hydroxy-11-oxo-olean-12-en-30-oate* (**20**)*.* Compound **20** was obtained from GA and *trans*-4-hydroxycinnamic acid ethyl ester as a white solid (548.1 mg, 85%); m.p. 195–196 °C; ^1^H-NMR (CDCl_3_): δ 0.69–0.72 (m, 1H, H-5), 0.81 (s, 3H, H-28), 0.86 (s, 3H, H-24), 0.94–0.98 (m, 1H, H-1’), 1.01 (s, 3H, H-23), 1.06–1.09 (m, 1H, H-15’), 1.14 (s, 3H, H-26), 1.14 (s, 3H, H-25), 1.19–1.21 (m, 1H, H-16’), 1.24–1.26 (m, 2H, H-22’ and H-21’), 1.34 (t, *J* = 7.2 Hz, 3H, CH_3_), 1.35 (s, 3H, H-29), 1.40 (s, 3H, H-27), 1.44–1.45 (m, 1H, H-22), 1.46–1.48 (m, 1H, H-7’), 1.49–1.53 (m, 1H, H-6’), 1.60–1.62 (m, 1H, H-6), 1.63 (dd, *J* = 13.2, 4.0 Hz, 1H, H-19’), 1.67–1.69 (m, 1H, H-2’), 1.72–1.74 (m, 1H, H-7), 1.75–1.78 (m, 1H, H-2), 1.86 (ddd, *J* = 14.4, 14.4, 5.2 Hz, 1H, H-16), 2.04–2.06 (m, 1H, H-21), 2.12–2.14 (m, 1H, H-15), 2.24 (dd, *J* = 13.2, 4.0 Hz, 1H, H-18), 2.35 (s, 1H, H-9), 2.43–2.45 (m, 1H, H-19), 2.78 (ddd, *J* = 13.2, 3.2, 3.2 Hz, 1H, H-1), 3.23 (dd, *J* = 10.8, 5.6 Hz, 1H, H-3), 4.27 (q, *J* = 7.2 Hz, 2H, CH_2_CH_3_), 5.68 (s, 1H, H-12), 6.40 (d, *J_trans_* = 16.0 Hz, 1H, H-β), 7.08 (d, *J* = 8.8 Hz, 2H, Bn-H-2 and 6), 7.55 (d, *J* = 8.8 Hz, 2H, Bn-H-3 and 5), 7.67 (d, *J_trans_* = 16.0 Hz, 1H, H-α); ^13^C-NMR (CDCl_3_): δ 200.11, 174.78, 168.70, 166.82, 162.12, 145.40, 132.12, 129.14, 128.61, 121.97, 118.33, 78.64, 61.76, 60.61, 54.86, 48.43, 45.33, 44.31, 43.16, 41.00, 39.07, 39.03, 37.67, 37.01, 32.69, 31.88, 31.06, 28.54, 28.04, 28.02, 27.21, 26.39, 26.34, 23.38, 18.61, 17.42, 16.30, 15.54, 14.27; ESI-MS: *m*/*z* = 645.31 [M + H]^+^. Anal. Calcd. for C_41_H_56_O_6_ (644.41): C, 76.36; H, 8.75%. Found: C, 76.31; H, 8.79%.

*Isoferulic acid methyl ester 3β-hydroxy-11-oxo-olean-12-en-30-oate* (**21**)*.* Compound **21** was obtained from GA and isoferulic acid methyl ester as a white solid (575 mg, 87%); m.p. 215–217 °C; ^1^H-NMR (CDCl_3_): δ 0.70–0.72 (m, 1H, H-5), 0.81 (s, 3H, H-28), 0.89 (s, 3H, H-24), 0.94–0.98 (m, 1H, H-1’), 1.01 (s, 3H, H-23), 1.06–1.09 (m, 1H, H-15’), 1.14 (s, 3H, H-26), 1.15 (s, 3H, H-25), 1.19–1.21 (m, 1H, H-16’), 1.24–1.26 (m, 2H, H-22’ and 21’), 1.36 (s, 3H, H-29), 1.40 (s, 3H, H-27), 1.44 (m, 1H, H-22), 1.47 (m, 1H, H-7’), 1.49 (m, 1H, H-6’), 1.62 (m, 1H, H-6), 1.64 (dd, 1H, *J* = 13.2, 4.0 Hz, H-19’), 1.67 (m, 1H, H-2’), 1.70 (m, 1H, H-7), 1.75 (m, 1H, H-2), 1.88 (ddd, 1H, *J* = 14.4, 14.4, 5.2 Hz, H-16), 2.06 (m, 1H, H-21), 2.10 (m, 1H, H-15), 2.13 (dd, 1H, *J* = 13.2, 4.0 Hz, H-18), 2.35 (s, 1H, H-9), 2.42 (m, 1H, H-19), 2.78 (ddd, 1H, *J* = 13.2, 3.2, 3.2 Hz, H-1), 3.23 (dd, 1H, *J* = 10.8, 5.6 Hz, H-3), 3.79 (s, 3H, COOCH_3_), 3.86 (s, 3H, OCH_3_), 5.70 (s, 1H, H-12), 6.30 (d, 1H, *J_trans_* = 16.0 Hz, H-β), 6.96 (d, 1H, *J* = 8.8 Hz, Bn-H-3), 7.17 (d, 1H, *J* = 2.0 Hz, Bn-H-6), 7.36 (dd, 1H, *J* = 8.8, 2.0 Hz, Bn-H-4), 7.61 (d, 1H, *J_trans_* = 16.0 Hz, H-α); ^13^C-NMR (CDCl_3_): δ 200.23, 174.38, 169.22, 167.43, 152.86, 143.66, 139.91, 128.42, 127.56, 127.40, 121.68, 116.24, 112.25, 78.63, 61.76, 55.81, 54.85, 51.62, 47.95, 45.32, 44.34, 43.12, 41.16, 39.07, 39.06, 37.42, 37.00, 32.69, 31.81, 31.15, 28.53, 28.29, 28.04, 27.20, 26.45, 26.36, 23.34, 18.63, 17.42, 16.31, 15.55; ESI-MS: *m*/*z* = 661.28 [M + H]^+^. Anal. Calcd. for C_41_H_56_O_7_ (660.40): C, 74.51; H, 8.54%. Found: C, 74.46; H, 8.56%.

*Isoferulic acid ethyl ester 3β-hydroxy-11-oxo-olean-12-en-30-oate* (**22**)*.* Compound **22** was obtained from GA and isoferulic acid ethyl ester as a white solid (567 mg, 84%); m.p. 210–213 °C; ^1^H-NMR (CDCl_3_): δ 0.69–0.72 (m, 1H, H-5), 0.81 (s, 3H, H-28 ), 0.88 (s, 3H, H-24), 0.94–0.98 (m, 1H, H-1’), 1.01 (s, 3H, H-23), 1.05–1.09 (m, 1H, H-15’), 1.14 (s, 3H, H-26), 1.15 (s, 3H, H-25), 1.19–1.21 (m, 1H, H-16’), 1.24–1.26 (m, 2H, H-22’ and 21’), 1.33 (t, 3H, *J* = 7.2 Hz, CH_3_), 1.36 (s, 3H, H-29), 1.40 (s, 3H, H-27), 1.44 (m, 1H, H-22), 1.47 (m, 1H, H-7’), 1.50 (m, 1H, H-6’), 1.62 (m, 1H, H-6), 1.64 (dd, 1H, *J* = 13.2, 4.0 Hz, H-19’), 1.67 (m, 1H, H-2’), 1.70 (m, 1H, H-7), 1.75 (m, 1H, H-2), 1.87 (ddd, 1H, *J* = 14.0, 14.0, 4.8 Hz, H-16), 2.06 (m, 1H, H-21), 2.10 (m, 1H, H-15), 2.13 (dd, 1H, *J* = 12.8, 3.6 Hz, H-18), 2.35 (s, 1H, H-9), 2.42 (m, 1H, H-19), 2.78 (ddd, 1H, *J* = 13.6, 3.6, 3.6 Hz, H-1), 3.23 (dd, 1H, *J* = 11.2, 5.2 Hz, H-3), 3.86 (s, 3H, OCH_3_), 4.25 (q, 2H, *J* = 7.2 Hz, CH_2_CH_3_), 5.70 (s, 1H, H-12), 6.30 (d, 1H, *J_trans_* = 16.0 Hz, H-β), 6.96 (d, 1H, *J* = 8.8 Hz, Bn-H-3), 7.18 (d, 1H, *J* = 2.0 Hz, Bn-H-6), 7.36 (dd, 1H, *J* = 8.8, 2.0 Hz, Bn-H-4), 7.60 (d, 1H, *J_trans_* = 16.0 Hz, H-α); ^13^C-NMR (CDCl_3_): δ 200.23, 174.38, 169.22, 167.43, 152.86, 143.66, 139.91, 128.42, 127.56, 127.40, 121.68, 116.24, 112.25, 78.63, 61.76, 55.81, 54.85, 51.62, 47.95, 45.32, 44.34, 43.12, 41.16, 39.07, 39.06, 37.42, 37.00, 32.69, 31.81, 31.15, 28.53, 28.29, 28.04, 27.20, 26.45, 26.36, 23.34, 18.63, 17.42, 16.31, 15.55; ESI-MS: *m*/*z* = 675.26 [M + H]^+^. Anal. Calcd. for C_42_H_58_O_7_ (674.42): C, 74.74; H, 8.66%. Found: C, 74.70; H, 8.69%.

*trans-3-Hydroxycinnamic acid methyl ester 3β-hydroxy-11-oxo-olean-12-en-30-oate* (**23**)*.* Compound **23** was obtained from GA and *trans*-3-hydroxycinnamic acid methyl ester as a white solid (511 mg, 81%); m.p. 188–190 °C; ^1^H-NMR (CDCl_3_): δ 0.72–0.75 (m, 1H, H-5), 0.83 (s, 3H, H-28), 0.90 (s, 3H, H-24), 0.97–1.00 (m, 1H, H-1’), 1.03 (s, 3H, H-23), 1.08–1.12 (m, 1H, H-15’), 1.16 (s, 3H, H-26), 1.17 (s, 3H, H-25), 1.20–1.23 (m, 1H, H-16’), 1.27–1.28 (m, 2H, H-22’ and 21’), 1.38 (s, 3H, H-29), 1.42 (s, 3H, H-27), 1.44 (m, 1H, H-22), 1.47 (m, 1H, H-7’), 1.50 (m, 1H, H-6’), 1.62 (m, 1H, H-6), 1.66 (dd, 1H, *J* = 13.2, 4.0 Hz, H-19’), 1.69 (m, 1H, H-2’), 1.74 (m, 1H, H-7), 1.77 (m, 1H, H-2), 1.89 (ddd, 1H, *J* = 14.8, 14.8, 5.6 Hz, H-16), 2.08 (m, 1H, H-21), 2.16 (m, 1H, H-15), 2.28 (dd, 1H, *J* = 13.6, 4.4 Hz, H-18), 2.37 (s, 1H, H-9), 2.43 (m, 1H, H-19), 2.81 (ddd, 1H, *J* = 12.8, 2.8, 2.8 Hz, H-1), 3.25 (dd, 1H, *J* = 10.4, 6.0 Hz, H-3), 3.84 (s, 3H, COOCH_3_), 5.70 (s, 1H, H-12), 6.46 (d, 1H, *J_trans_* = 16.0 Hz, H-β), 7.09 (dt, 1H, *J* = 6.8, 2.4 Hz, Bn-H-6), 7.21 (s, 1H, Bn-H-2), 7.42 (s, 1H, Bn-H-4), 7.44 (t, 1H, *J* = 7.6 Hz, Bn-H-5), 7.70 (d, 1H, *J_trans_* = 16.0 Hz, H-α); ^13^C-NMR (CDCl_3_): δ 200.07, 174.89, 168.68, 167.04, 151.12, 145.65, 135.96, 129.89, 128.61, 125.61, 123.24, 120.69, 118.92, 78.65, 61.77, 54.86, 51.74, 48.36, 45.33, 44.28, 43.15, 40.97, 39.07, 39.04, 37.74, 37.02, 32.69, 31.88, 31.08, 28.58, 28.07, 28.05, 27.22, 26.39, 26.34, 23.39, 18.62, 17.42, 16.30, 15.54; ESI-MS: *m*/*z* = 631.23 [M + H]^+^. Anal. Calcd. for C_40_H_54_O_6_ (630.39): C, 76.16; H, 8.63%. Found: C, 76.11; H, 8.67%.

*trans-3-Hydroxycinnamic acid ethyl ester 3β-hydroxy-11-oxo-olean-12-en-30-oate* (**24**)*.* Compound **24** was obtained from GA and *trans*-3-hydroxycinnamic acid ethyl ester as a white solid (561 mg, 87%); m.p. 173–175 °C; ^1^H-NMR (CDCl_3_): δ 0.72–0.75 (m, 1H, H-5), 0.83 (s, 3H, H-28), 0.90 (s, 3H, H-24), 0.97–1.00 (m, 1H, H-1’), 1.03 (s, 3H, H-23), 1.08–1.12 (m, 1H, H-15’), 1.16 (s, 3H, H-26), 1.17 (s, 3H, H-25), 1.20–1.23 (m, 1H, H-16’), 1.27–1.28 (m, 2H, H-22’ and 21’), 1.36 (t, 3H, *J* = 7.2 Hz, CH_3_), 1.38 (s, 3H, H-29), 1.42 (s, 3H, H-27), 1.44 (m, 1H, H-22), 1.47 (m, 1H, H-7’), 1.50 (m, 1H, H-6’), 1.62 (m, 1H, H-6), 1.66 (dd, 1H, *J* = 13.2, 4.0 Hz, H-19’), 1.69 (m, 1H, H-2’), 1.74 (m, 1H, H-7), 1.77 (m, 1H, H-2), 1.89 (ddd, 1H, *J* = 14.4, 14.4, 5.2 Hz, H-16), 2.08 (m, 1H, H-21), 2.16 (m, 1H, H-15), 2.28 (dd, 1H, *J* = 13.2, 4.0 Hz, H-18), 2.37 (s, 1H, H-9), 2.43 (m, 1H, H-19), 2.81 (ddd, 1H, *J* = 13.2, 3.2, 3.2 Hz, H-1), 3.25 (dd, 1H, *J* = 10.8, 5.6 Hz, H-3), 4.29 (q, 2H, *J* = 7.2 Hz, CH_2_CH_3_), 5.70 (s, 1H, H-12), 6.46 (d, 1H, *J_trans_* = 16.0 Hz, H-β), 7.08 (dt, 1H, *J* = 6.8, 2.4 Hz, Bn-H-6), 7.22 (s, 1H, Bn-H-2), 7.42 (s, 1H, Bn-H-4), 7.44 (t, 1H, *J* = 7.6 Hz, Bn-H-5), 7.68 (d, 1H, *J_trans_* = 16.0 Hz, H-α); ^13^C-NMR (CDCl_3_): δ 200.15, 174.76, 168.73, 166.96, 151.00, 145.48, 135.85, 129.73, 128.50, 125.49, 123.02, 120.57, 118.80, 78.56, 61.69, 60.32, 54.72, 48.21, 45.23, 44.20, 43.02, 40.86, 38.94, 38.90, 37.62, 36.95, 32.56, 31.73, 30.98, 28.40, 27.97, 27.93, 27.12, 26.25, 26.21, 23.23, 18.52, 17.30, 16.23, 15.46, 14.30; ESI-MS: *m*/*z* = 667.29 [M + Na]^+^. Anal. Calcd. for C_41_H_56_O_6_ (644.41): C, 76.36; H, 8.75%. Found: C, 76.29; H, 8.78%.

### 3.5. General Method for Synthesizing Compouds **25**–**40**

The protected amino acid (*N*-Boc-l-methionine or *N*-Boc-l-selenomethionine, 0.5 mmol) was dissolved in dry DCM and stirred at 0 °C for 5 min. Then EDCI (115 mg, 0.6 mmol) and DMAP (12 mg, 0.1 mmol) were added to the solution, and the mixture was stirred at 0 °C for 1 h. The appropriate GA derivative **17**–**24** (0.5 mmol) was then added to the mixture. After stirring at 0 °C for another 1 h, the reaction mixture was then stirred at room temperature for 12 h. The organic layer was washed with 1 M HCl solution and concentrated *in vacuo*. The residue was purified by column chromatography on silica gel with ethyl acetate/petroleum to yield pure compound **25**–**40**.

*Ferulic acid methyl ester 3β-(Boc-l-methionine)-11-oxo-olean-12-en-30-oate* (**25**). Obtained from **17** and *N*-Boc-l-methionine as a colourless powder (357 mg, 80%); m.p. 120–123 °C; ^1^H-NMR (CDCl_3_): δ 0.82–0.84 (m, 1H, H-5), 0.89 (s, 3H, H-28), 0.91 (s, 3H, H-24), 0.92 (s, 3H, H-23), 1.07–1.11 (m, 1H, H-15’), 1.14–1.16 (m, 1H, H-1’), 1.17 (s, 3H, H-26), 1.19 (s, 3H, H-25), 1.22–1.25 (m, 1H, H-16’), 1.27–1.31 (m, 2H, H-22’ and 21’), 1.38 (s, 3H, H-29), 1.41 (s, 3H, H-27), 1.47 (s, 9H, Boc-CH_3_), 1.49–1.51 (m, 1H, H-22), 1.53–1.55 (m, 1H, H-7’), 1.57–1.59 (m, 1H, H-6’), 1.62–1.64 (m, 1H, H-6), 1.67 (dd, *J* = 13.6, 4.4 Hz, 1H, H-19’), 1.70–1.72 (m, 1H, H-2’), 1.73–1.74 (m, 1H, H-7), 1.77–1.80 (m, 1H, H-2), 1.89 (ddd, *J* = 14.8, 14.8, 5.6 Hz, 1H, H-16), 2.03–2.05 (m, 1H, H-21), 2.09–2.11 (m, 1H, H-15), 2.12 (s, 3H, SCH_3_), 2.15 (dd, *J* = 13.6, 4.4 Hz, 1H, H-18), 2.38 (s, 1H, H-9), 2.43–2.45 (m, 1H, H-19), 2.57–2.60 (m, 2H, SCH_2_), 2.83 (ddd, *J* = 13.6, 2.8, 2.8 Hz, 1H, H-1), 3.83 (s, 3H, COOCH_3_), 3.88 (s, 3H, OCH_3_), 4.80 (dd, *J* = 11.2, 5.2 Hz, 1H, H-3), 5.14 (t, *J* = 8.4 Hz, 1H, COOCH_3_), 5.73 (s, 1H, H-12), 6.42 (d, *J_trans_* = 16.0 Hz, 1H, H-β), 7.02 (d, *J* = 8.0 Hz, 1H, Bn-H-5), 7.13 (s, 1H, Bn-H-3), 7.15 (d, *J* = 8.0 Hz, 1H, Bn-H-6), 7.68 (d, *J_trans_* = 16.0 Hz, 1H, H-α); ^13^C-NMR (CDCl_3_): δ 199.82, 174.40, 171.90, 169.23, 166.85, 154.87, 151.38, 149.94, 145.89, 128.40, 123.02, 121.15, 118.31, 113.49, 109.10, 81.93, 79.69, 61.54, 55.69, 54.94, 52.73, 51.67, 48.00, 45.33, 44.47, 43.11, 41.18, 38.06, 38.02, 37.40, 36.86, 32.64, 32.10, 31.81, 31.15, 29.63, 28.47, 28.26, 28.09, 28.04, 26.40, 26.34, 23.48, 23.28, 18.59, 17.32, 16.74, 16.30, 15.36; ESI-MS: *m*/*z* = 892.25 [M + H]^+^. Anal. Calcd. for C_51_H_73_NO_10_S (891.50): C, 68.66; H, 8.25; N, 1.57; S, 3.59%. Found: C, 68.60; H, 8.28; N, 1.54; S, 3.60%.

*Ferulic acid methyl ester 3β-(Boc-l-selenomethionine)-11-oxo-olean-12-en-30-oate* (**26**). Obtained from **17** and *N*-Boc-l-selenomethionine as a colourless powder (389 mg, 83%); m.p. 125–127 °C; ^1^H-NMR (CDCl_3_): δ 0.82–0.85 (m, 1H, H-5), 0.90 (s, 3H, H-28), 0.91 (s, 3H, H-24), 0.92 (s, 3H, H-23), 1.07–1.11 (m, 1H, H-15’), 1.14–1.16 (m, 1H, H-1’), 1.18 (s, 3H, H-26), 1.19 (s, 3H, H-25), 1.23–1.25 (m, 1H, H-16’), 1.27–1.30 (m, 2H, H-22’ and 21’), 1.38 (s, 3H, H-29), 1.42 (s, 3H, H-27), 1.46 (s, 9H, Boc-CH_3_), 1.49–1.52 (m, 1H, H-22), 1.54–1.56 (m, 1H, H-7’), 1.58–1.60 (m, 1H, H-6’), 1.62–1.65 (m, 1H, H-6), 1.67 (dd, *J* = 13.6, 4.4 Hz, 1H, H-19’), 1.70–1.72 (m, 1H, H-2’), 1.73–1.76 (m, 1H, H-7), 1.77–1.79 (m, 1H, H-2), 1.88 (ddd, *J* = 14.8, 14.8, 5.6 Hz, 1H, H-16), 1.89-1.92 (m, 1H, H-21), 1.98–2.00 (m, 1H, H-15), 2.02 (s, 3H, SeCH_3_), 2.12 (dd, *J* = 13.6, 4.4 Hz, 1H, H-18), 2.39 (s, 1H, H-9), 2.44–2.47 (m, 1H, H-19), 2.58–2.61 (m, 2H, SeCH_2_), 2.83 (ddd, *J* = 13.6, 2.8, 2.8 Hz, 1H, H-1), 3.84 (s, 3H, COOCH_3_), 3.89 (s, 3H, OCH_3_), 4.59 (dd, *J* = 11.2, 5.2 Hz, 1H, H-3), 5.12 (t, *J* = 8.4 Hz, 1H, CHCOO), 5.73 (s, 1H, H-12), 6.42 (d, *J_trans_* = 16.0 Hz, 1H, H-β), 7.02 (d, *J* = 8.0 Hz, 1H, Bn-H-5), 7.13 (s, 1H, Bn-H-3), 7.15 (d, *J* = 8.0 Hz, 1H, Bn-H-6), 7.68 (d, *J_trans_* = 16.0 Hz, 1H, H-α); ^13^C-NMR (CDCl_3_): δ 199.82, 174.40, 171.82, 169.17, 166.85, 154.90, 151.40, 149.94, 145.89, 128.40, 123.02, 121.15, 118.32, 113.49, 109.10, 82.12, 79.72, 61.54, 55.70, 54.97, 52.71, 51.67, 48.02, 45.34, 44.47, 43.15, 41.20, 38.10, 38.06, 37.43, 36.86, 32.65, 32.07, 31.84, 31.15, 29.64, 28.50, 28.27, 28.12, 28.02, 26.41, 26.35, 23.45, 23.30, 18.60, 17.32, 16.75, 16.30, 15.37; ESI-MS: *m*/*z* = 962.18 [M + Na]^+^. Anal. Calcd. for C_51_H_73_NO_10_Se (939.44): C, 65.23; H, 7.84; N, 1.49%. Found: C, 65.18; H, 7.89; N, 1.46%.

*Ferulic acid ethyl ester 3β-(Boc-l-methionine)-11-oxo-olean-12-en-30-oate* (**27**). Obtained from **18** and *N*-Boc-l-methionine as a colourless powder (357.8 mg, 79%); m.p. 111–113 °C; ^1^H-NMR (CDCl_3_): δ 0.80–0.83 (m, 1H, H-5), 0.88 (s, 3H, H-28), 0.89 (s, 3H, H-24), 0.90 (s, 3H, H-23), 1.05–1.08 (m, 1H, H-15’), 1.11–1.13 (m, 1H, H-1’), 1.15 (s, 3H, H-26), 1.17 (s, 3H, H-25), 1.20–1.23 (m, 1H, H-16’), 1.26–1.28 (m, 2H, H-22’ and 21’), 1.34 (t, *J* = 7.2 Hz, 3H, CH_3_), 1.35 (s, 3H, H-29), 1.39 (s, 3H, H-27), 1.45 (s, 9H, Boc-CH_3_), 1.48–1.50 (m, 1H, H-22), 1.51–1.54 (m, 1H, H-7’), 1.58–1.60 (m, 1H, H-6’), 1.61–1.63 (m, 1H, H-6), 1.68 (dd, *J* = 13.6, 4.4 Hz, 1H, H-19’), 1.71–1.73 (m, 1H, H-2’), 1.75–1.77 (m, 1H, H-7), 1.78–1.81 (m, 1H, H-2), 1.89 (ddd, *J* = 14.4, 14.4, 5.2 Hz, 1H, H-16), 2.04–2.06 (m, 1H, H-21), 2.08–2.09 (m, 1H, H-15), 2.10 (s, 3H, SCH_3_), 2.15 (dd, *J* = 13.2, 4.0 Hz, 1H, H-18), 2.37–2.39 (m, 1H, H-9), 2.41–2.44 (m, 1H, H-19), 2.55–2.57 (m, 2H, SCH_2_), 2.81 (ddd, *J* = 14.0, 3.2, 3.2 Hz, 1H, H-1), 3.86 (s, 3H, OCH_3_), 4.27 (q, *J* = 7.2 Hz, 2H, CH_2_CH_3_), 4.57 (dd, *J* = 11.6, 4.8 Hz, 1H, H-3), 5.12 (t, *J* = 8.4 Hz, 1H, CHCOO), 5.71 (s, 1H, H-12), 6.39 (d, *J_trans_* = 16.0 Hz, 1H, H-β), 7.00 (d, *J* = 7.6 Hz, 1H, Bn-H-5), 7.11 (s, 1H, Bn-H-3), 7.12 (d, *J* = 7.6 Hz, 1H, Bn-H-6), 7.65 (d, *J_trans_* = 16.0 Hz, 1H, H-α); ^13^C-NMR (CDCl_3_): δ 199.92, 174.28, 171.90, 169.32, 166.77, 155.10, 151.33, 149.92, 145.86, 128.38, 123.00, 121.10, 118.30, 113.49, 109.10, 82.03, 79.80, 61.60, 60.50, 55.67, 54.94, 52.71, 48.00, 45.32, 44.36, 43.13, 41.16, 38.05, 38.04, 37.36, 36.83, 32.61, 32.12, 31.81, 31.16, 29.63, 28.47, 28.26, 28.12, 28.04, 26.45, 26.34, 23.50, 23.28, 18.62, 17.30, 16.74, 16.32, 15.37, 14.26; ESI-MS: *m*/*z* = 928.27 [M + Na]^+^. Anal. Calcd. for C_52_H_75_NO_10_S (905.51): C, 68.92; H, 8.34; N, 1.55; S, 3.54%. Found: C, 68.86; H, 8.37; N, 1.51; S, 3.57%.

*Ferulic acid ethyl ester 3β-(Boc-l-selenomethionine)-11-oxo-olean-12-en-30-oate* (**28**)*.* Obtained from **18** and N-Boc-l-selenomethionine as a colourless powder (390 mg, 82%); m.p. 118–120 °C; ^1^H-NMR (CDCl_3_): δ 0.82–0.85 (m, 1H, H-5), 0.90 (s, 3H, H-28), 0.91 (s, 3H, H-24), 0.92 (s, 3H, H-23), 1.07–1.10 (m, 1H, H-15’), 1.11–1.13 (m, 1H, H-1’), 1.17 (s, 3H, H-26), 1.19 (s, 3H, H-25), 1.22–1.26 (m, 1H, H-16’), 1.27–1.30 (m, 2H, H-22’ and 21’), 1.37 (t, *J* = 7.2 Hz, 3H, CH_3_), 1.38 (s, 3H, H-29), 1.42 (s, 3H, H-27), 1.47 (s, 9H, Boc-CH_3_), 1.49–1.51 (m, 1H, H-22), 1.53–1.56 (m, 1H, H-7’), 1.58–1.60 (m, 1H, H-6’), 1.63–1.65 (m, 1H, H-6), 1.67 (dd, *J* = 13.6, 4.4 Hz, 1H, H-19’), 1.73–1.75 (m, 1H, H-2’), 1.76–1.78 (m, 1H, H-7), 1.80–1.83 (m, 1H, H-2), 1.89 (ddd, *J* = 14.4, 14.4, 5.2 Hz, 1H, H-16), 1.97–1.99 (m, 1H, H-21), 2.00–2.01 (m, 1H, H-15), 2.02 (s, 3H, SeCH_3_), 2.12 (dd, *J* = 13.2, 4.0 Hz, 1H, H-18), 2.39 (s, 1H, H-9), 2.43–2.46 (m, 1H, H-19), 2.59–2.61 (m, 2H, SeCH_2_), 2.83 (ddd, *J* = 14.0, 3.2, 3.2 Hz, 1H, H-1), 3.88 (s, 3H, OCH_3_), 4.29 (q, *J* = 7.2 Hz, 2H, CH_2_CH_3_), 4.59 (dd, *J* = 11.6, 4.8 Hz, 1H, H-3), 5.13 (t, *J* = 8.4 Hz, 1H, CHCOO), 5.73 (s, 1H, H-12), 6.41 (d, *J_trans_* = 16.0 Hz, 1H, H-β), 7.02 (d, *J* = 8.0 Hz, 1H, Bn-H-5), 7.13 (s, 1H, Bn-H-3), 7.14 (d, *J* = 8.0 Hz, 1H, Bn-H-6), 7.67 (d, *J_trans_* = 16.0 Hz, 1H, H-α); ^13^C-NMR (CDCl_3_): δ 199.92, 174.28, 171.83, 169.24, 166.77, 155.12, 151.36, 149.95, 145.86, 128.38, 123.03, 121.10, 118.30, 113.49, 109.10, 82.09, 79.83, 61.60, 60.50, 55.68, 54.95, 52.68, 48.02, 45.32, 44.36, 43.14, 41.18, 38.08, 38.04, 37.37, 36.83, 32.62, 32.10, 31.81, 31.16, 29.63, 28.49, 28.27, 28.15, 28.03, 26.46, 26.36, 23.47, 23.29, 18.64, 17.30, 16.76, 16.32, 15.37, 14.26; ESI-MS: *m*/*z* = 978.12 [M + Na]^+^. Anal. Calcd. for C_52_H_75_NO_10_Se (953.11): C, 65.53; H, 7.93; N, 1.47%. Found: C, 65.46; H, 7.98; N, 1.44%.

*trans-4-Hydroxycinnamic acid methyl ester 3β-(Boc-l-methionine)-11-oxo-olean-12-en-30-oate* (**29**). Obtained from **19** and *N*-Boc-l-methionine as a colourless powder (362 mg, 84%); m.p. 107–109 °C; ^1^H-NMR (CDCl_3_): δ 0.82-0.85 (m, 1H, H-5), 0.88 (s, 3H, H-28), 0.91 (s, 3H, H-24), 0.92 (s, 3H, H-23), 1.08-1.11 (m, 1H, H-15’), 1.13–1.15 (m, 1H, H-1’), 1.16 (s, 3H, H-26), 1.18 (s, 3H, H-25), 1.23–1.25 (m, 1H, H-16’), 1.27–1.31 (m, 2H, H-22’ and 21’), 1.37 (s, 3H, H-29), 1.42 (s, 3H, H-27), 1.47 (s, 9H, Boc-CH_3_), 1.48–1.50 (m, 1H, H-22), 1.51–1.53 (m, 1H, H-7’), 1.60–1.62 (m, 1H, H-6’), 1.64–1.66 (m, 1H, H-6), 1.67 (dd, *J* = 13.6, 4.4 Hz, 1H, H-19’), 1.74–1.76 (m, 1H, H-2’), 1.77–1.78 (m, 1H, H-7), 1.80–1.83 (m, 1H, H-2), 1.89 (ddd, *J* = 15.2, 15.2, 6.0 Hz, 1H, H-16), 2.06–2.08 (m, 1H, H-21), 2.10–2.11 (m, 1H, H-15), 2.12-2.15 (s, 3H, SCH_3_), 2.26 (dd, *J* = 14.0, 4.8 Hz, 1H, H-18), 2.39 (s, 1H, H-9), 2.43–2.46 (m, 1H, H-19), 2.57–2.60 (m, 2H, SCH_2_), 2.83 (ddd, *J* = 13.2, 2.4, 2.4 Hz, 1H, H-1), 3.83 (s, 3H, COOCH_3_), 4.59 (dd, *J* = 10.8, 5.6 Hz, 1H, H-3), 5.14 (t, *J* = 9.2 Hz, 1H, CHCOO), 5.70 (s, 1H, H-12), 6.43 (d, *J_trans_* = 16.0 Hz, 1H, H-β), 7.10 (d, *J* = 8.4 Hz, 2H, Bn-H-2 and 6), 7.57 (d, *J* = 8.4 Hz, 2H, Bn-H-3 and 5), 7.70 (d, *J_trans_* = 16.0 Hz, 1H, H-α); ^13^C-NMR (CDCl_3_): δ 199.80, 174.83, 172.02, 168.76, 166.90, 162.24, 155.22, 145.41, 132.17, 129.13, 128.58, 121.92, 118.41, 81.98, 79.85, 61.73, 54.94, 52.88, 51.83, 48.43, 45.36, 44.39, 43.24, 41.10, 38.16, 38.12, 37.78, 37.15, 32.66, 32.23, 31.88, 31.13, 29.87, 28.54, 28.18, 28.16, 28.06, 26.43, 26.40, 23.52, 23.39, 18.65, 17.33, 16.74, 16.42, 15.42; ESI-MS: *m*/*z* = 884.22 [M + Na]^+^. Anal. Calcd. for C_50_H_71_NO_9_S (861.48): C, 69.65; H, 8.30; N, 1.62; S, 3.72%. Found: C, 69.59; H, 8.33; N, 1.58; S, 3.75%.

*trans-4-Hydroxycinnamic acid methyl ester 3β-(Boc-l-selenomethionine)-11-oxo-olean-12-en-30-oate* (**30**)*.* Obtained from **19** and *N*-Boc-l-selenomethionine as a colourless powder (363 mg, 80%); m.p. 115–118 °C; ^1^H-NMR (400 MHz, CDCl_3_): δ 0.82–0.85 (m, 1H, H-5), 0.88 (s, 3H, H-28), 0.91 (s, 3H, H-24), 0.92 (s, 3H, H-23), 1.08–1.11 (m, 1H, H-15’), 1.13–1.15 (m, 1H, H-1’), 1.17 (s, 3H, H-26), 1.19 (s, 3H, H-25), 1.22–1.26 (m, 1H, H-16’), 1.28–1.31 (m, 2H, H-22’ and 21’), 1.38 (s, 3H, H-29), 1.42 (s, 3H, H-27), 1.47 (s, 9H, Boc-CH_3_), 1.49–1.51 (m, 1H, H-22), 1.53–1.55 (m, 1H, H-7’), 1.60–1.62 (m, 1H, H-6’), 1.64–1.66 (m, 1H, H-6), 1.68 (dd, *J* = 13.6, 4.4 Hz, 1H, H-19’), 1.74–1.76 (m, 1H, H-2’), 1.77–1.79 (m, 1H, H-7), 1.81–1.84 (m, 1H, H-2), 1.90 (ddd, *J* = 15.2, 15.2, 6.0 Hz, 1H, H-16), 1.97–1.99 (m, 1H, H-21), 2.00–2.01 (m, 1H, H-15), 2.02 (s, 3H, SeCH_3_), 2.26 (dd, *J* = 14.0, 4.8 Hz, 1H, H-18), 2.39 (s, 1H, H-9), 2.43–2.46 (m, 1H, H-19), 2.58–2.60 (m, 2H, SeCH_2_), 2.84 (ddd, *J* = 13.2, 2.4, 2.4 Hz, 1H, H-1), 3.83 (s, 3H, COOCH_3_), 4.59 (dd, *J* = 10.8, 5.6 Hz, 1H, H-3), 5.13 (t, *J* = 9.2 Hz, 1H, CHCOO), 5.70 (s, 1H, H-12), 6.43 (d, *J_trans_* = 16.0 Hz, 1H, H-β), 7.10 (d, *J* = 8.4 Hz, 2H, Bn-H-2 and 6), 7.57 (d, *J* = 8.4 Hz, 2H, Bn-H-3 and 5), 7.71 (d, *J_trans_* = 16.0 Hz, 1H, H-α); ^13^C-NMR (CDCl_3_): δ 199.80, 174.83, 171.90, 168.67, 166.90, 162.25, 155.25, 145.41, 132.16, 129.13, 128.58, 121.92, 118.44, 82.07, 79.88, 61.73, 54.98, 52.85, 51.83, 48.46, 45.36, 44.39, 43.27, 41.13, 38.20, 38.12, 37.80, 37.15, 32.62, 32.20, 31.90, 31.15, 29.87, 28.57, 28.20, 28.18, 28.04, 26.44, 26.42, 23.49, 23.40, 18.67, 17.33, 16.75, 16.42, 15.42; ESI-MS: *m*/*z* = 932.16 [M + Na]^+^. Anal. Calcd. for C_50_H_71_NO_9_Se (909.43): C, 66.06; H, 7.87; N, 1.54%. Found: C, 66.00; H, 7.91; N, 1.51%.

*trans-4-Hydroxycinnamic acid ethyl ester 3β-(Boc-l-methionine)-11-oxo-olean-12-en-30-oate* (**31**). Obtained from **20** and *N*-Boc-l-methionine as a colourless powder (359 mg, 82%); m.p. 102–104 °C; ^1^H-NMR (CDCl_3_): δ 0.82–0.85 (m, 1H, H-5), 0.88 (s, 3H, H-28), 0.91 (s, 3H, H-24), 0.92 (s, 3H, H-23), 1.08–1.11 (m, 1H, H-15’), 1.13–1.15 (m, 1H, H-1’), 1.16 (s, 3H, H-26), 1.18 (s, 3H, H-25), 1.23–1.26 (m, 1H, H-16’), 1.28–1.30 (m, 2H, H-22’ and 21’), 1.36 (t, *J* = 7.2 Hz, 3H, CH_3_), 1.37 (s, 3H, H-29), 1.42 (s, 3H, H-27), 1.47 (s, 9H, Boc-CH_3_), 1.49–1.50 (m, 1H, H-22), 1.51–1.54 (m, 1H, H-7’), 1.61–1.63 (m, 1H, H-6’), 1.64–1.66 (m, 1H, H-6), 1.67 (dd, *J* = 13.6, 4.4 Hz, 1H, H-19’), 1.73–1.75 (m, 1H, H-2’), 1.77–1.79 (m, 1H, H-7), 1.80–1.83 (m, 1H, H-2), 1.88 (ddd, *J* = 14.8, 14.8, 5.6 Hz, 1H, H-16), 2.07–2.09 (m, 1H, H-21), 2.10–2.11 (m, 1H, H-15), 2.12 (s, 3H, SCH_3_), 2.27 (dd, *J* = 13.6, 4.4 Hz, 1H, H-18), 2.39 (s, 1H, H-9), 2.43–2.46 (m, 1H, H-19), 2.57–2.59 (m, 2H, SCH_2_), 2.83 (ddd, *J* = 13.6, 2.8, 2.8 Hz, 1H, H-1), 4.29 (q, *J* = 7.2 Hz, 2H, CH_2_CH_3_), 4.59 (dd, *J* = 11.2, 5.2 Hz, 1H, H-3), 5.14 (t, *J* = 9.2 Hz, 1H, CHCOO), 5.70 (s, 1H, H-12), 6.42 (d, *J_trans_* = 16.0 Hz, 1H, H-β), 7.10 (d, *J* = 8.4 Hz, 2H, Bn-H-2 and 6), 7.57 (d, *J* = 8.4 Hz, 2H, Bn-H-3 and 5), 7.69 (d, *J_trans_* = 16.0 Hz, 1H, H-α); ^13^C-NMR (CDCl_3_): δ 199.83, 174.76, 171.93, 168.81, 166.79, 162.11, 155.21, 145.37, 132.13, 129.14, 128.56, 121.95, 118.38, 82.00, 79.83, 61.62, 60.49, 54.94, 52.85, 48.44, 45.33, 44.31, 43.16, 40.99, 38.07, 38.04, 37.67, 36.84, 32.60, 32.25, 31.88, 31.06, 29.84, 28.53, 28.26, 28.12, 28.01, 26.39, 26.33, 23.47, 23.33, 18.61, 17.28, 16.75, 16.33, 15.41, 14.26; ESI-MS: *m*/*z* = 898.25 [M + Na]^+^. Anal. Calcd. for C_51_H_73_NO_9_S (875.50): C, 69.91; H, 8.40; N, 1.60; S, 3.66%. Found: C, 69.84; H, 8.43; N, 1.55; S, 3.73%.

*trans-4-Hydroxycinnamic acid ethyl ester 3β-(Boc-l-selenomethionine)-11-oxo-olean-12-en-30-oate* (**32**). Obtained from **20** and *N*-Boc-l-selenomethionine as a colourless powder (383 mg, 83%); m.p. 110–112 °C; ^1^H-NMR (CDCl_3_): δ 0.82–0.85 (m, 1H, H-5), 0.89 (s, 3H, H-28), 0.91 (s, 3H, H-24), 0.92 (s, 3H, H-23), 1.08–1.11 (m, 1H, H-15’), 1.13–1.15 (m, 1H, H-1’), 1.17 (s, 3H, H-26), 1.19 (s, 3H, H-25), 1.23–1.26 (m, 1H, H-16’), 1.28–1.30 (m, 2H, H-22’ and 21’), 1.37 (t, *J* = 7.2 Hz, 3H, CH_3_), 1.38 (s, 3H, H-29), 1.42 (s, 3H, H-27), 1.47 (s, 9H, Boc-CH_3_), 1.48–1.50 (m, 1H, H-22), 1.51–1.53 (m, 1H, H-7’), 1.60–1.62 (m, 1H, H-6’), 1.64–1.66 (m, 1H, H-6), 1.67 (dd, *J* = 13.6, 4.4 Hz, 1H, H-19’), 1.73–1.75 (m, 1H, H-2’), 1.77–1.79 (m, 1H, H-7), 1.81–1.84 (m, 1H, H-2), 1.89 (ddd, *J* = 14.8, 14.8, 5.6 Hz, 1H, H-16), 1.92–1.94 (m, 1H, H-21), 1.97–1.99 (m, 1H, H-15), 2.02 (s, 3H, SeCH_3_), 2.26 (dd, *J* = 13.6, 4.4 Hz, 1H, H-18), 2.39 (s, 1H, H-9), 2.46–2.49 (m, 1H, H-19), 2.57–2.60 (m, 2H, SeCH_2_), 2.84 (ddd, *J* = 13.6, 2.8, 2.8 Hz, 1H, H-1), 4.29 (q, *J* = 7.2 Hz, 2H, CH_2_CH_3_), 4.59 (dd, *J* = 11.2, 5.2 Hz, 1H, H-3), 5.13 (t, *J* = 9.2 Hz, 1H, CHCOO), 5.70 (s, 1H, H-12), 6.42 (d, *J_trans_* = 16.0 Hz, 1H, H-β), 7.10 (d, *J* = 8.4 Hz, 2H, Bn-H-2 and 6), 7.57 (d, *J* = 8.4 Hz, 2H, Bn-H-3 and 5), 7.70 (d, *J_trans_* = 16.0 Hz, 1H, H-α); ^13^C-NMR (CDCl_3_): δ 199.83, 174.75, 171.84, 168.78, 166.79, 162.13, 155.25, 145.37, 132.16, 129.14, 128.56, 121.94, 118.38, 82.09, 79.87, 61.62, 60.47, 54.96, 52.80, 48.49, 45.33, 44.36, 43.15, 41.03, 38.05, 38.04, 37.65, 36.78, 32.60, 32.23, 31.88, 31.00, 29.82, 28.59, 28.30, 28.15, 27.96, 26.41, 26.36, 23.42, 23.35, 18.60, 17.28, 16.77, 16.33, 15.40, 14.28; ESI-MS: *m*/*z* = 946.23 [M + Na]^+^. Anal. Calcd. for C_51_H_73_NO_9_Se (923.45): C, 66.36; H, 7.97; N, 1.52%. Found: C, 66.33; H, 8.02; N, 1.48%.

*Isoferulic acid methyl ester 3β-(Boc-l-methionine)-11-oxo-olean-12-en-30-oate* (**33**)*.* Obtained from **21** and *N*-Boc-l-methionine as a colourless powder (361 mg, 81%); m.p. 117–119 °C; ^1^H-NMR (CDCl_3_): δ 0.82–0.85 (m, 1H, H-5), 0.89 (s, 3H, H-28), 0.90 (s, 3H, H-24), 0.92 (s, 3H, H-23), 1.07–1.11 (m, 1H, H-15’), 1.14–1.16 (m, 1H, H-1’), 1.17 (s, 3H, H-26), 1.18 (s, 3H, H-25), 1.22–1.26 (m, 1H, H-16’), 1.28–1.29 (m, 2H, H-22’ and 21’), 1.38 (s, 3H, H-29), 1.42 (s, 3H, H-27), 1.46 (s, 9H, Boc-CH_3_), 1.49 (m, 1H, H-22), 1.53 (m, 1H, H-7’), 1.58 (m, 1H, H-6’), 1.62 (m, 1H, H-6), 1.67 (dd, 1H, *J* = 13.6, 4.4 Hz, H-19’), 1.73 (m, 1H, H-2’), 1.77 (m, 1H, H-7), 1.80 (m, 1H, H-2), 1.88 (ddd, 1H, *J* = 14.8, 14.8, 5.2 Hz, H-16), 2.06 (m, 1H, H-21), 2.10 (m, 1H, H-15), 2.12 (s, 3H, SCH_3_), 2.15 (dd, 1H, *J* = 13.2, 4.4 Hz, H-18), 2.39 (s, 1H, H-9), 2.44 (m, 1H, H-19), 2.57 (m, 2H, SCH_2_), 2.83 (ddd, 1H, *J* = 13.2, 2.8, 2.8 Hz, H-1), 3.81 (s, 3H, COOCH_3_), 3.88 (s, 3H, OCH_3_), 4.59 (dd, 1H, *J* = 11.2, 5.2 Hz, H-3), 5.14 (t, 1H, *J* = 8.4 Hz, CHCOO), 5.72 (s, 1H, H-12), 6.32 (d, 1H, *J_trans_* = 16.0 Hz, H-β), 6.98 (d, 1H, *J* = 8.4 Hz, Bn-H-3), 7.19 (d, 1H, *J* = 2.0 Hz, Bn-H-6), 7.38 (dd, 1H, *J* = 8.4, 2.0 Hz, Bn-H-4), 7.63 (d, 1H, *J_trans_* = 16.0 Hz, H-α); ^13^C-NMR (CDCl_3_): δ 199.95, 174.34, 171.83, 169.03, 166.96, 154.82, 152.83, 143.64, 138.11, 128.42, 127.43, 127.38, 121.70, 116.21, 112.26, 81.90, 79.66, 61.56, 55.71, 54.87, 52.69, 51.53, 47.93, 45.29, 44.36, 43.03, 41.02, 38.95, 38.85, 37.84, 36.90, 32.63, 32.12, 31.83, 31.12, 29.60, 28.47, 28.33, 28.07, 28.05, 26.35, 26.28, 23.50, 23.13, 18.57, 17.30, 16.73, 16.25, 15.37; ESI-MS: *m*/*z* = 914.28 [M + Na]^+^. Anal. Calcd. for C_51_H_73_NO_10_S (891.50): C, 68.66; H, 8.25; N, 1.57; S, 3.59%. Found: C, 68.60; H, 8.31; N, 1.53; S, 3.63%.

*Isoferulic acid methyl ester 3β-(Boc-l-selenomethionine)-11-oxo-olean-12-en-30-oate* (**34**)*.* Obtained from **21** and *N*-Boc-l-selenomethionine as a colourless powder (375 mg, 80%); m.p. 124–125 °C; ^1^H-NMR (CDCl_3_): δ 0.82–0.85 (m, 1H, H-5), 0.89 (s, 3H, H-28), 0.90 (s, 3H, H-24), 0.92 (s, 3H, H-23), 1.07–1.11 (m, 1H, H-15’), 1.14–1.16 (m, 1H, H-1’), 1.18 (s, 3H, H-26), 1.19 (s, 3H, H-25), 1.22–1.26 (m, 1H, H-16’), 1.28–1.29 (m, 2H, H-22’ and 21’), 1.38 (s, 3H, H-29), 1.42 (s, 3H, H-27), 1.47 (s, 9H, Boc-CH_3_), 1.49 (m, 1H, H-22), 1.52 (m, 1H, H-7’), 1.60 (m, 1H, H-6’), 1.62 (m, 1H, H-6), 1.67 (dd, 1H, *J* = 13.6, 4.4 Hz, H-19’), 1.73 (m, 1H, H-2’), 1.77 (m, 1H, H-7), 1.80 (m, 1H, H-2), 1.88 (ddd, 1H, *J* = 14.8, 14.8, 5.2 Hz, H-16), 1.93 (m, 1H, H-21), 2.00 (m, 1H, H-15), 2.02 (s, 3H, SeCH_3_), 2.13 (dd, 1H, *J* = 13.2, 4.4 Hz, H-18), 2.39 (s, 1H, H-9), 2.44 (m, 1H, H-19), 2.58 (m, 2H, SeCH_2_), 2.83 (ddd, 1H, *J* = 13.2, 2.8, 2.8 Hz, H-1), 3.81 (s, 3H, COOCH_3_), 3.88 (s, 3H, OCH_3_), 4.59 (dd, 1H, *J* = 11.2, 5.2 Hz, H-3), 5.12 (t, 1H, *J* = 8.4 Hz, CHCOO), 5.73 (s, 1H, H-12), 6.32 (d, 1H, *J_trans_* = 16.0 Hz, H-β), 6.98 (d, 1H, *J* = 8.4 Hz, Bn-H-3), 7.19 (d, 1H, *J* = 2.0 Hz, Bn-H-6), 7.38 (dd, 1H, *J* = 8.4, 2.0 Hz, Bn-H-4), 7.63 (d, 1H, *J_trans_* = 16.0 Hz, H-α); ^13^C-NMR (CDCl_3_): δ 199.95, 174.34, 171.75, 169.00, 166.97, 154.85, 152.85, 143.64, 138.11, 128.42, 127.43, 127.38, 121.70, 116.21, 112.26, 81.90, 79.70, 61.56, 55.73, 54.88, 52.67, 51.53, 47.96, 45.31, 44.36, 43.06, 41.05, 39.02, 38.85, 37.86, 36.90, 32.65, 32.04, 31.85, 31.13, 29.58, 28.52, 28.33, 28.10, 28.03, 26.37, 26.30, 23.47, 23.10, 18.59, 17.30, 16.74, 16.26, 15.39; ESI-MS: *m*/*z* = 962.12 [M + Na]^+^. Anal. Calcd. for C_51_H_73_NO_10_Se (939.44): C, 65.23; H, 7.84; N, 1.49%. Found: C, 65.15; H, 7.89; N, 1.43%.

*Isoferulic acid ethyl ester 3β-(Boc-l-methionine)-11-oxo-olean-12-en-30-oate* (**35**)*.* Obtained from **22** and *N*-Boc-l-methionine as a colourless powder (376 mg, 83%); m.p. 111–114 °C; ^1^H-NMR (CDCl_3_): δ 0.80–0.83 (m, 1H, H-5), 0.88 (s, 3H, H-28), 0.89 (s, 3H, H-24), 0.90 (s, 3H, H-23), 1.05–1.09 (m, 1H, H-15’), 1.14–1.16 (m, 1H, H-1’), 1.16 (s, 3H, H-26), 1.17 (s, 3H, H-25), 1.20–1.24 (m, 1H, H-16’), 1.26–1.28 (m, 2H, H-22’ and 21’), 1.33 (t, 3H, *J* = 7.2 Hz, CH_3_), 1.36 (s, 3H, H-29), 1.40 (s, 3H, H-27), 1.45 (s, 9H, Boc-CH_3_), 1.48 (m, 1H, H-22), 1.51 (m, 1H, H-7’), 1.58 (m, 1H, H-6’), 1.61 (m, 1H, H-6), 1.64 (dd, 1H, *J* = 13.6, 4.4 Hz, H-19’), 1.72 (m, 1H, H-2’), 1.75 (m, 1H, H-7), 1.78 (m, 1H, H-2), 1.88 (ddd, 1H, *J* = 14.4, 14.4, 5.2 Hz, H-16), 2.05 (m, 1H, H-21), 2.10 (m, 1H, H-15), 2.10 (s, 3H, SCH_3_), 2.14 (dd, 1H, *J* = 13.2, 4.0 Hz, H-18), 2.37 (s, 1H, H-9), 2.43 (m, 1H, H-19), 2.55 (m, 2H, SCH_2_), 2.81 (ddd, 1H, *J* = 14.0, 3.2, 3.2 Hz, H-1), 3.86 (s, 3H, OCH_3_), 4.25 (q, 2H, *J* = 7.2 Hz, CH_2_CH_3_), 4.57 (dd, 1H, *J* = 11.6, 4.8 Hz, H-3), 5.12 (t, 1H, *J* = 8.4 Hz, CHCOO), 5.71 (s, 1H, H-12), 6.30 (d, 1H, *J_trans_* = 16.0 Hz, H-β), 6.96 (d, 1H, *J* = 8.4 Hz, Bn-H-3), 7.18 (d, 1H, *J* = 2.0 Hz, Bn-H-6), 7.36 (dd, 1H, *J* = 8.4, 2.0 Hz, Bn-H-4), 7.60 (d, 1H, *J_trans_* = 16.0 Hz, H-α); ^13^C-NMR (CDCl_3_): δ 200.13, 174.23, 171.83, 169.13, 166.88, 155.04, 152.80, 143.61, 138.08, 128.40, 127.40, 127.38, 121.65, 116.20, 112.26, 81.93, 79.78, 61.65, 60.65, 55.70, 54.87, 52.70, 47.93, 45.27, 44.27, 43.05, 41.05, 38.96, 38.85, 37.80, 36.87, 32.60, 32.15, 31.83, 31.14, 29.61, 28.47, 28.33, 28.10, 28.05, 26.37, 26.30, 23.52, 23.13, 18.59, 17.29, 16.73, 16.28, 15.38, 14.23; ESI-MS: *m*/*z* = 928.24 [M + Na]^+^. Anal. Calcd. for C_52_H_75_NO_10_S (905.51): C, 68.92; H, 8.34; N, 1.55; S, 3.54%. Found: C, 68.87; H, 8.39; N, 1.51; S, 3.56%.

*Isoferulic acid methyl ester 3β-(Boc-l-selenomethionine)-11-oxo-olean-12-en-30-oate* (**36**)*.* Obtained from **22** and *N*-Boc-l-selenomethionine as a colourless powder (405 mg, 85%); m.p. 119–121 °C; ^1^H-NMR (CDCl_3_): δ 0.80–0.83 (m, 1H, H-5), 0.88 (s, 3H, H-28), 0.89 (s, 3H, H-24), 0.90 (s, 3H, H-23), 1.05–1.09 (m, 1H, H-15’), 1.12–1.14 (m, 1H, H-1’), 1.16 (s, 3H, H-26), 1.17 (s, 3H, H-25), 1.20–1.24 (m, 1H, H-16’), 1.26–1.28 (m, 2H, H-22’ and 21’), 1.33 (t, 3H, *J* = 7.2 Hz, CH_3_), 1.36 (s, 3H, H-29), 1.40 (s, 3H, H-27), 1.45 (s, 9H, Boc-CH_3_), 1.48 (m, 1H, H-22), 1.51 (m, 1H, H-7’), 1.59 (m, 1H, H-6’), 1.62 (m, 1H, H-6), 1.64 (dd, 1H, *J* = 13.6, 4.4 Hz, H-19’), 1.71 (m, 1H, H-2’), 1.75 (m, 1H, H-7), 1.78 (m, 1H, H-2), 1.88 (ddd, 1H, *J* = 14.4, 14.4, 5.2 Hz, H-16), 1.96 (m, 1H, H-21), 1.98(m, 1H, H-15), 2.00 (s, 3H, SeCH_3_), 2.10 (dd, 1H, *J* = 13.2, 4.0 Hz, H-18), 2.37 (s, 1H, H-9), 2.43 (m, 1H, H-19), 2.55 (m, 2H, SeCH_2_), 2.81 (ddd, 1H, *J* = 14.0, 3.2, 3.2 Hz, H-1), 3.86 (s, 3H, OCH_3_), 4.25 (q, 2H, *J* = 7.2 Hz, CH_2_CH_3_), 4.57 (dd, 1H, *J* = 11.6, 4.8 Hz, H-3), 5.10 (t, 1H, *J* = 8.4 Hz, CHCOO), 5.71 (s, 1H, H-12), 6.30 (d, 1H, *J_trans_* = 16.0 Hz, H-β), 6.96 (d, 1H, *J* = 8.8 Hz, Bn-H-3), 7.18 (d, 1H, *J* = 2.0 Hz, Bn-H-6), 7.36 (dd, 1H, *J* = 8.8, 2.0 Hz, Bn-H-4), 7.60 (d, 1H, *J_trans_* = 16.0 Hz, H-α); ^13^C-NMR (CDCl_3_): δ 200.14 174.23, 171.77, 169.05, 166.88, 155.06, 152.83, 143.62, 138.08, 128.40, 127.40, 127.38, 121.65, 116.20, 112.26, 81.93, 79.80, 61.65, 60.65, 55.72, 54.89, 52.66, 47.94, 45.27, 44.27, 43.06, 41.08, 38.96, 38.86, 37.82, 36.87, 32.61, 32.12, 31.83, 31.14, 29.61, 28.48, 28.31, 28.12, 28.04, 26.39, 26.30, 23.49, 23.15, 18.61, 17.29, 16.75, 16.28, 15.38, 14.23; ESI-MS: *m*/*z* = 976.15 [M + Na]^+^. Anal. Calcd. for C_52_H_75_NO_10_Se (953.11): C, 65.53; H, 7.93; N, 1.47%. Found: C, 65.48; H, 8.00; N, 1.43%.

*trans-3-Hydroxycinnamic acid methyl ester 3β-(Boc-l-methionine)-11-oxo-olean-12-en-30-oate* (**37**)*.* Obtained from **23** and *N*-Boc-l-methionine as a colourless powder (349 mg, 81%); m.p. 109–111 °C; ^1^H-NMR (CDCl_3_): δ 0.82–0.85 (m, 1H, H-5), 0.90 (s, 3H, H-28), 0.91 (s, 3H, H-24), 0.92 (s, 3H, H-23), 1.08–1.12 (m, 1H, H-15’), 1.13–1.15 (m, 1H, H-1’), 1.17(s, 3H, H-26), 1.19 (s, 3H, H-25), 1.23–1.25 (m, 1H, H-16’), 1.26–1.28 (m, 2H, H-22’ and 21’), 1.39 (s, 3H, H-29), 1.42 (s, 3H, H-27), 1.47 (s, 9H, Boc-CH_3_), 1.50 (m, 1H, H-22), 1.53 (m, 1H, H-7’), 1.61 (m, 1H, H-6’), 1.64 (m, 1H, H-6), 1.67 (dd, 1H, *J* = 13.6, 4.4 Hz, H-19’), 1.74 (m, 1H, H-2’), 1.77 (m, 1H, H-7), 1.80 (m, 1H, H-2), 1.89 (ddd, 1H, *J* = 15.2, 15.2, 6.0 Hz, H-16), 2.07 (m, 1H, H-21), 2.10 (m, 1H, H-15), 2.13 (s, 3H, SCH_3_), 2.28 (dd, 1H, *J* = 14.0, 4.8 Hz, H-18), 2.39 (s, 1H, H-9), 2.46 (m, 1H, H-19), 2.57 (m, 2H, SCH_2_), 2.84 (ddd, 1H, *J* = 13.2, 2.4, 2.4 Hz, H-1), 3.83 (s, 3H, COOCH_3_), 4.59 (dd, 1H, *J* = 10.8, 5.6 Hz, H-3), 5.14 (t, 1H, *J* = 9.2 Hz, CHCOO), 5.71 (s, 1H, H-12), 6.46 (d, 1H, *J_trans_* = 16.0 Hz, H-β), 7.09 (dt, 1H, *J* = 6.8, 2.4 Hz, Bn-H-6), 7.21 (s, 1H, Bn-H-2), 7.42 (s, 1H, Bn-H-4), 7.44 (t, 1H, *J* = 7.6 Hz, Bn-H-5), 7.70 (d, 1H, *J_trans_* = 16.0 Hz, H-α); ^13^C-NMR (CDCl_3_): δ 199.80, 174.87, 171.91, 168.82, 167.02, 155.24, 151.11, 143.61, 135.95, 129.88, 128.57, 125.50, 123.22, 120.67, 118.89, 81.99, 79.82, 61.61, 54.92, 52.85, 51.76, 48.36, 45.32, 44.28, 43.15, 40.94, 38.57, 38.07, 37.72, 36.84, 32.59, 32.23, 31.88, 31.07, 29.84, 28.57, 28.26, 28.12, 28.07, 26.37, 26.32, 23.53, 23.33, 18.61, 17.28, 16.75, 16.31, 15.41; ESI-MS: *m*/*z* = 884.27 [M + Na]^+^. Anal. Calcd. for C_50_H_71_NO_9_S (861.48): C, 69.65; H, 8.30; N, 1.62; S, 3.72%. Found: C, 69.61; H, 8.35; N, 1.57; S, 3.78%.

*trans-3-Hydroxycinnamic acid methyl ester 3β-(Boc-l-selenomethionine)-11-oxo-olean-12-en-30-oate* (**38**)*.* Obtained from **23** and *N*-Boc-l-selenomethionine as a colourless powder (377 mg, 83%); m.p. 112–115 °C; ^1^H-NMR (CDCl_3_): δ 0.82–0.85 (m, 1H, H-5), 0.90 (s, 3H, H-28), 0.91 (s, 3H, H-24), 0.92 (s, 3H, H-23), 1.08–1.11 (m, 1H, H-15’), 1.13–1.15 (m, 1H, H-1’), 1.17(s, 3H, H-26), 1.19 (s, 3H, H-25), 1.23–1.25 (m, 1H, H-16’), 1.26–1.28 (m, 2H, H-22’ and 21’), 1.39 (s, 3H, H-29), 1.42 (s, 3H, H-27), 1.47 (s, 9H, Boc-CH_3_), 1.50 (m, 1H, H-22), 1.53 (m, 1H, H-7’), 1.61 (m, 1H, H-6’), 1.65 (m, 1H, H-6), 1.67 (dd, 1H, *J* = 13.6, 4.4 Hz, H-19’), 1.74 (m, 1H, H-2’), 1.77 (m, 1H, H-7), 1.80 (m, 1H, H-2), 1.89 (ddd, 1H, *J* = 15.2, 15.2, 6.0 Hz, H-16), 1.97 (m, 1H, H-21), 2.01 (m, 1H, H-15), 2.02 (s, 3H, SeCH_3_), 2.16 (dd, 1H, *J* = 14.0, 4.8 Hz, H-18), 2.39 (s, 1H, H-9), 2.46 (m, 1H, H-19), 2.58 (m, 2H, SeCH_2_), 2.84 (ddd, 1H, *J* = 13.2, 2.4, 2.4 Hz, H-1), 3.83 (s, 3H, COOCH_3_), 4.59 (dd, 1H, *J* = 10.8, 5.6 Hz, H-3), 5.13 (t, 1H, *J* = 9.2 Hz, CHCOO), 5.71 (s, 1H, H-12), 6.46 (d, 1H, *J_trans_* = 16.0 Hz, H-β), 7.09 (dt, 1H, *J* = 6.8, 2.4 Hz, Bn-H-6), 7.21 (s, 1H, Bn-H-2), 7.42 (s, 1H, Bn-H-4), 7.44 (t, 1H, *J* = 7.6 Hz, Bn-H-5), 7.70 (d, 1H, *J_trans_* = 16.0 Hz, H-α); ^13^C-NMR (CDCl_3_): δ 199.80, 174.87, 171.83, 168.78, 167.02, 155.26, 151.12, 143.61, 135.96, 129.88, 128.57, 125.50, 123.22, 120.67, 118.90, 82.06, 79.84, 61.61, 54.93, 52.83, 51.76, 48.37, 45.32, 44.28, 43.16, 40.95, 38.59, 38.07, 37.73, 36.84, 32.60, 32.21, 31.88, 31.07, 29.84, 28.58, 28.27, 28.14, 28.06, 26.38, 26.33, 23.49, 23.34, 18.62, 17.28, 16.76, 16.31, 15.41; ESI-MS: *m*/*z* = 932.22 [M + Na]^+^. Anal. Calcd. for C_50_H_71_NO_9_Se (909.43): C, 66.06; H, 7.87; N, 1.54%. Found: C, 66.01; H, 7.91; N, 1.52%.

*trans-3-Hydroxycinnamic acid ethyl ester 3β-(Boc-l-methionine)-11-oxo-olean-12-en-30-oate* (**39**)*.* Obtained from **24** and *N*-Boc-l-methionine as a colourless powder (341 mg, 78%); m.p. 107–108 °C; ^1^H-NMR (CDCl_3_): δ 0.82–0.85 (m, 1H, H-5), 0.90 (s, 3H, H-28), 0.91 (s, 3H, H-24), 0.92 (s, 3H, H-23), 1.08–1.12 (m, 1H, H-15’), 1.13–1.15 (m, 1H, H-1’), 1.17(s, 3H, H-26), 1.19 (s, 3H, H-25), 1.23–1.25 (m, 1H, H-16’), 1.26–1.28 (m, 2H, H-22’ and 21’), 1.36 (t, 3H, *J* = 7.2 Hz, CH_3_), 1.38 (s, 3H, H-29), 1.42 (s, 3H, H-27), 1.47 (s, 9H, Boc-CH_3_), 1.50 (m, 1H, H-22), 1.53 (m, 1H, H-7’), 1.61 (m, 1H, H-6’), 1.64 (m, 1H, H-6), 1.67 (dd, 1H, *J* = 13.6, 4.4 Hz, H-19’), 1.74 (m, 1H, H-2’), 1.77 (m, 1H, H-7), 1.80 (m, 1H, H-2), 1.89 (ddd, 1H, *J* = 14.8, 14.8, 5.6 Hz, H-16), 2.07 (m, 1H, H-21), 2.10 (m, 1H, H-15), 2.13 (s, 3H, SCH_3_), 2.28 (dd, 1H, *J* = 13.6, 4.4 Hz, H-18), 2.39 (s, 1H, H-9), 2.46 (m, 1H, H-19), 2.56 (m, 2H, SCH_2_), 2.84 (ddd, 1H, *J* = 13.6, 2.8, 2.8 Hz, H-1), 4.29 (q, 2H, *J* = 7.2 Hz, CH_2_CH_3_), 4.59 (dd, 1H, *J* = 11.2, 5.2 Hz, H-3), 5.15 (t, 1H, *J* = 9.2 Hz, CHCOO), 5.71 (s, 1H, H-12), 6.46 (d, 1H, *J_trans_* = 16.0 Hz, H-β), 7.08 (dt, 1H, *J* = 6.8, 2.4 Hz, Bn-H-6), 7.22 (s, 1H, Bn-H-2), 7.42 (s, 1H, Bn-H-4), 7.44 (t, 1H, *J* = 7.6 Hz, Bn-H-5), 7.69 (d, 1H, *J_trans_* = 16.0 Hz, H-α); ^13^C-NMR (CDCl_3_): δ 199.92, 174.72, 171.91, 168.98, 166.93, 155.64, 150.97, 143.48, 135.84, 129.72, 128.45, 125.38, 123.00, 120.54, 118.78, 82.10, 79.97, 61.73, 60.45, 54.92, 52.83, 48.36, 45.32, 44.10, 43.16, 40.85, 38.58, 38.06, 37.69, 36.82, 32.57, 32.26, 31.85, 31.09, 29.85, 28.57, 28.26, 28.14, 28.07, 26.45, 26.32, 23.54, 23.33, 18.63, 17.27, 16.75, 16.32, 15.41, 14.30; ESI-MS: *m*/*z* = 898.30 [M + Na]^+^. Anal. Calcd. for C_51_H_73_NO_9_S (875.50): C, 69.91; H, 8.40; N, 1.60; S, 3.66%. Found: C, 69.87; H, 8.46; N, 1.54; S, 3.70%.

*trans-3-Hydroxycinnamic acid ethyl ester 3β-(Boc-l-selenomethionine)-11-oxo-olean-12-en-30-oate* (**40**)*.* Obtained from **24** and *N*-Boc-l-selenomethionine as a colourless powder (364 mg, 79%); m.p. 109–112 °C; ^1^H-NMR (CDCl_3_): δ 0.82–0.85 (m, 1H, H-5), 0.90 (s, 3H, H-28), 0.91 (s, 3H, H-24), 0.92 (s, 3H, H-23), 1.08–1.11 (m, 1H, H-15’), 1.13–1.15 (m, 1H, H-1’), 1.17(s, 3H, H-26), 1.19 (s, 3H, H-25), 1.23–1.25 (m, 1H, H-16’), 1.26–1.28 (m, 2H, H-22’ and 21’), 1.36 (t, 3H, *J* = 7.2 Hz, CH_3_), 1.38 (s, 3H, H-29), 1.42 (s, 3H, H-27), 1.47 (s, 9H, Boc-CH_3_), 1.50 (m, 1H, H-22), 1.53 (m, 1H, H-7’), 1.61 (m, 1H, H-6’), 1.64 (m, 1H, H-6), 1.70 (dd, 1H, *J* = 13.6, 4.4 Hz, H-19’), 1.74 (m, 1H, H-2’), 1.77 (m, 1H, H-7), 1.80 (m, 1H, H-2), 1.90 (ddd, 1H, *J* = 14.8, 14.8, 5.6 Hz, H-16), 1.98 (m, 1H, H-21), 2.01 (m, 1H, H-15), 2.02 (s, 3H, SeCH_3_), 2.29 (dd, 1H, *J* = 13.6, 4.4 Hz, H-18), 2.39 (s, 1H, H-9), 2.46 (m, 1H, H-19), 2.58 (m, 2H, SeCH_2_), 2.84 (ddd, 1H, *J* = 13.6, 2.8, 2.8 Hz, H-1), 4.29 (q, 2H, *J* = 7.2 Hz, CH_2_CH_3_), 4.59 (dd, 1H, *J* = 11.2, 5.2 Hz, H-3), 5.13 (t, 1H, *J* = 9.2 Hz, CHCOO), 5.71 (s, 1H, H-12), 6.46 (d, 1H, *J_trans_* = 16.0 Hz, H-β), 7.08 (dt, 1H, *J* = 6.8, 2.4 Hz, Bn-H-6), 7.22 (s, 1H, Bn-H-2), 7.42 (s, 1H, Bn-H-4), 7.44 (t, 1H, *J* = 7.6 Hz, Bn-H-5), 7.68 (d, 1H, *J_trans_* = 16.0 Hz, H-α); ^13^C-NMR (CDCl_3_): δ 199.92, 174.72, 171.82, 168.93, 166.93, 155.67, 150.98, 143.48, 135.86, 129.72, 128.45, 125.36, 123.03, 120.51, 118.78, 82.17, 80.00, 61.73, 60.44, 54.95, 52.82, 48.38, 45.32, 44.13, 43.19, 40.87, 38.57, 38.09, 37.68, 36.79, 32.58, 32.26, 31.84, 31.03, 29.85, 28.60, 28.29, 28.15, 28.03, 26.46, 26.36, 23.51, 23.30, 18.62, 17.27, 16.78, 16.32, 15.41, 14.31; ESI-MS: *m*/*z* = 946.26 [M + Na]^+^. Anal. Calcd. for C_51_H_73_NO_9_Se (923.45): C, 66.36; H, 7.97; N, 1.52%. Found: C, 66.33; H, 8.01; N, 1.47%.

### 3.6. General Method for Synthesizing Compouds **41**–**56**

The Boc-protected compounds **25**–**40** (0.25 mmol) were dissolved in dry DCM. After saturation with dry hydrogen chloride gas for 10 min, stirring at room temperature was then continued for 24 h. After completion of the reaction (as monitored by TLC), the resulting precipitate was filtered and washed with ethyl acetate until no parent substance can be detected; analytical samples were obtained by re-crystallization to obtain pure compounds **41**–**56**.

*Ferulic acid methyl ester 3β-(l-methionine)-11-oxo-olean-12-en-30-oate hydrochloride* (**41**). Obtained from **25** as a white solid (155 mg, 81%); m.p. 203–205 °C; ^1^H-NMR (DMSO-*d*_6_): δ 0.71–0.74 (m, 1H, H-5), 0.83 (s, 3H, H-28), 0.84 (s, 3H, H-24), 0.85 (s, 3H, H-23), 0.90–0.93 (m, 1H, H-15’), 1.00–1.03 (m, 1H, H-1’), 1.07 (s, 3H, H-26), 1.08 (s, 3H, H-25), 1.12–1.14 (m, 1H, H-16’), 1.20–1.24 (m, 2H, H-22’ and 21’), 1.32 (s, 3H, H-29), 1.41 (s, 3H, H-27), 1.43–1.45 (m, 1H, H-22), 1.48–1.50 (m, 1H, H-7’), 1.51–1.52 (m, 1H, H-6’), 1.53–1.55 (m, 1H, H-6), 1.62 (dd, *J* = 13.2, 4.0 Hz, 1H, H-19’), 1.67–1.69 (m, 1H, H-2’), 1.71–1.73 (m, 1H, H-7), 1.75–1.78 (m, 1H, H-2), 1.86 (ddd, *J* = 14.8, 14.8, 6.0 Hz, 1H, H-16), 1.91–1.93 (m, 1H, H-21), 1.95–1.97 (m, 1H, H-15), 2.04 (s, 3H, SCH_3_), 2.28–2.31 (m, 1H, H-18), 2.43 (s, 1H, H-9), 2.54–2.56 (m, 1H, H-19), 2.57–2.60 (m, 2H, SCH_2_), 2.64 (ddd, *J* = 13.2, 3.2, 3.2 Hz, 1H, H-1), 3.74 (s, 3H, COOCH_3_), 3.83 (s, 3H, OCH_3_), 4.47 (dd, *J* = 11.2, 5.2 Hz, 1H, H-3), 5.46 (s, 1H, H-12), 6.72 (d, *J_trans_* = 16.0 Hz, 1H, H-β), 7.11 (d, *J* = 8.0 Hz, 1H, Bn-H-5), 7.33 (dd, *J* = 8.0, 1.6 Hz, 1H, Bn-H-3), 7.55 (d, *J* = 1.6 Hz, 1H, Bn-H-6), 7.67 (d, *J_trans_* = 16.0 Hz, 1H, H-α); ^13^C-NMR (DMSO-*d*_6_): δ 199.91, 174.52, 173.90, 169.23, 166.87, 151.38, 149.96, 145.89, 128.40, 123.02, 121.15, 118.31, 113.51, 109.11, 81.45, 61.59, 55.69, 54.98, 52.84, 51.69, 48.03, 45.37, 44.50, 43.11, 41.28, 38.10, 38.04, 37.40, 36.86, 32.65, 32.12, 31.83, 31.25, 29.69, 28.52, 28.29, 28.10, 26.40, 26.38, 23.58, 23.30, 18.69, 17.42, 16.74, 16.33, 15.40; ESI-MS: *m*/*z* = 792.22 [M + H]^+^. Anal. Calcd. for C_46_H_66_ClNO_8_S (791.44): C, 66.68; H, 8.03; N, 1.69; S, 3.87%. Found: C, 66.63; H, 8.07; N, 1.66; S, 3.90%.

*Ferulic acid methyl ester 3β-(l-selenomethionine)-11-oxo-olean-12-en-30-oate hydrochloride* (**42**). Obtained from **26** as a white solid (156 mg, 77%); m.p. 210–213 °C; ^1^H-NMR (DMSO-*d*_6_): δ 0.71–0.74 (m, 1H, H-5), 0.83 (s, 3H, H-28), 0.84 (s, 3H, H-24), 0.85 (s, 3H, H-23), 0.91–0.94 (m, 1H, H-15’), 1.00–1.04 (m, 1H, H-1’), 1.07 (s, 3H, H-26), 1.08 (s, 3H, H-25), 1.12–1.15 (m, 1H, H-16’), 1.21–1.24 (m, 2H, H-22’ and 21’), 1.32 (s, 3H, H-29), 1.41 (s, 3H, H-27), 1.43–1.45 (m, 1H, H-22), 1.49–1.51 (m, 1H, H-7’), 1.52–1.53 (m, 1H, H-6’), 1.54–1.57 (m, 1H, H-6), 1.63 (dd, *J* = 13.2, 4.0 Hz, 1H, H-19’), 1.67–1.69 (m, 1H, H-2’), 1.71–1.73 (m, 1H, H-7), 1.76–1.78 (m, 1H, H-2), 1.86 (ddd, *J* = 14.8, 14.8, 6.0 Hz, 1H, H-16), 1.88–1.90 (m, 1H, H-21), 1.92–1.93 (m, 1H, H-15), 1.94 (s, 3H, SeCH_3_), 2.28–2.31 (m, 1H, H-18), 2.43 (s, 1H, H-9), 2.54–2.56 (m, 1H, H-19), 2.57–2.59 (m, 2H, SeCH_2_), 2.64 (ddd, *J* = 13.2, 3.2, 3.2 Hz, 1H, H-1), 3.74 (s, 3H, COOCH_3_), 3.84 (s, 3H, OCH_3_), 4.48 (dd, *J* = 11.2, 5.2 Hz, 1H, H-3), 5.46 (s, 1H, H-12), 6.72 (d, *J_trans_* = 16.0 Hz, 1H, H-β), 7.11 (d, *J* = 8.0 Hz, 1H, Bn-H-5), 7.33 (dd, *J* = 8.0, 1.6 Hz, 1H, Bn-H-3), 7.55 (d, *J* = 1.6 Hz, 1H, Bn-H-6), 7.67 (d, *J_trans_* = 16.0 Hz, 1H, H-α); ^13^C-NMR (DMSO-*d_6_*): δ 199.92, 174.47, 173.82, 169.27, 166.85, 151.40, 149.94, 145.90, 128.42, 123.02, 121.16, 118.32, 113.49, 109.10, 82.42, 61.54, 55.72, 55.00, 52.81, 51.67, 48.02, 45.34, 44.54, 43.15, 41.30, 38.06, 38.00, 37.43, 36.88, 32.65, 32.17, 31.84, 31.28, 29.74, 28.50, 28.27, 28.12, 26.43, 26.36, 23.55, 23.32, 18.71, 17.43, 16.75, 16.34, 15.38; ESI-MS: *m*/*z* = 840.26 [M + H]^+^. Anal. Calcd. for C_46_H_66_ClNO_8_Se (839.39): C, 63.11; H, 7.60; N, 1.60%. Found: C, 63.03; H, 7.65; N, 1.54%.

*Ferulic acid ethyl ester 3β-(l-methionine)-11-oxo-olean-12-en-30-oate hydrochloride* (**43**). Obtained from **27** as a white solid (142 mg, 73%); m.p. 191–194 °C; ^1^H-NMR (DMSO-*d*_6_): δ 0.74–0.78 (m, 1H, H-5), 0.83 (s, 3H, H-28), 0.84 (s, 3H, H-24), 0.85 (s, 3H, H-23), 0.90–0.93 (m, 1H, H-15’), 1.00–1.02 (m, 1H, H-1’), 1.07 (s, 3H, H-26), 1.08 (s, 3H, H-25), 1.12–1.14 (m, 1H, H-16), 1.17–1.23 (m, 2H, H-22’ and 21’), 1.27 (t, *J* = 7.2 Hz, 3H, CH_3_), 1.31 (s, 3H, H-29), 1.40 (s, 3H, H-27), 1.45–1.47 (m, 1H, H-22), 1.48–1.50 (m, 1H, H-7’), 1.51–1.52 (m, 1H, H-6’), 1.54–1.57 (m, 1H, H-6), 1.63 (dd, *J* = 13.2, 4.0 Hz, 1H, H-19’), 1.67–1.69 (m, 1H, H-2’), 1.70–1.73 (m, 1H, H-7), 1.79–1.81 (m, 1H, H-2), 1.86 (ddd, *J* = 14.4, 14.4, 5.6 Hz, 1H, H-16), 1.92-1.93 (m, 1H, H-21), 1.94–1.96 (m, 1H, H-15), 2.03 (s, 3H, SCH_3_), 2.28–2.31 (m, 1H, H-18), 2.42 (s, 1H, H-9), 2.54–2.55 (m, 1H, H-19), 2.56–2.59 (m, 2H, SCH_2_), 2.63 (ddd, *J* = 13.6, 3.6, 3.6 Hz, 1H, H-1), 3.83 (s, 3H, OCH_3_), 4.20 (q, *J* = 7.2 Hz, 2H, CH_2_CH_3_), 4.47 (dd, *J* = 11.6, 4.8 Hz, 1H, H-3), 5.46 (s, 1H, H-12), 6.72 (d, *J_trans_* = 16.0 Hz, 1H, H-β), 7.10 (d, *J* = 8.0 Hz, 1H, Bn-H-5), 7.31 (dd, *J* = 8.0, 1.6 Hz, 1H, Bn-H-3), 7.55 (d, *J* = 1.6 Hz, 1H, Bn-H-6), 7.64 (d, *J_trans_* = 16.0 Hz, 1H, H-α); ^13^C-NMR (DMSO-*d*_6_): δ 200.02, 174.37, 174.03, 169.45, 166.78, 151.33, 149.94, 145.86, 128.38, 123.03, 121.10, 118.30, 113.47, 109.12, 81.43, 61.60, 60.50, 55.67, 54.94, 53.52, 48.02, 45.33, 44.36, 43.15, 41.26, 38.07, 38.05, 37.36, 36.83, 32.63, 32.14, 31.79, 31.21, 29.72, 28.47, 28.37, 28.15, 26.45, 26.34, 23.63, 23.28, 18.75, 17.47, 16.72, 16.39, 15.46, 14.26; ESI-MS: *m*/*z* = 828.30 [M + Na]^+^. Anal. Calcd. for C_47_H_68_ClNO_8_S (805.46): C, 67.00; H, 8.13; N, 1.66; S, 3.81%. Found: C, 66.95; H, 8.20; N, 1.61; S, 3.85%.

*Ferulic acid ethyl ester 3β-(l-selenomethionine)-11-oxo-olean-12-en-30-oate hydrochloride* (**44**). Obtained from **28** as a white solid (160 mg, 78%); m.p. 205–207 °C; ^1^H-NMR (DMSO-*d*_6_): δ 0.70–0.74 (m, 1H, H-5), 0.83 (s, 3H, H-28), 0.84 (s, 3H, H-24), 0.85 (s, 3H, H-23), 0.91–0.93 (m, 1H, H-15’), 1.00–1.03 (m, 1H, H-1’), 1.07 (s, 3H, H-26), 1.08 (s, 3H, H-25), 1.12–1.14 (m, 1H, H-16’), 1.20–1.24 (m, 2H, H-22’ and 21’), 1.27 (t, *J* = 7.2 Hz, 3H, CH_3_), 1.32 (s, 3H, H-29), 1.41 (s, 3H, H-27), 1.46–1.48 (m, 1H, H-22), 1.49–1.50 (m, 1H, H-7’), 1.51–1.52 (m, 1H, H-6’), 1.54–1.56 (m, 1H, H-6), 1.65 (dd, *J* = 13.2, 4.0 Hz, 1H, H-19’), 1.68-1.69 (m, 1H, H-2’), 1.70–1.73 (m, 1H, H-7), 1.79–1.82 (m, 1H, H-2), 1.86 (ddd, *J* = 14.4, 14.4, 5.6 Hz, 1H, H-16), 1.89–1.90 (m, 1H, H-21), 1.91–1.92 (m, 1H, H-15), 1.93 (s, 3H, SeCH_3_), 2.28–2.31 (m, 1H, H-18), 2.43 (s, 1H, H-9), 2.54–2.56 (m, 1H, H-19), 2.57–2.60 (m, 2H, SeCH_2_), 2.63 (ddd, *J* = 13.6, 3.6, 3.6 Hz, 1H, H-1), 3.83 (s, 3H, OCH_3_), 4.20 (q, *J* = 7.2 Hz, 2H, CH_2_CH_3_), 4.47 (dd, *J* = 11.6, 4.8 Hz, 1H, H-3), 5.46 (s, 1H, H-12), 6.72 (d, *J_trans_* = 16.0 Hz, 1H, H-β), 7.11 (d, *J* = 8.0 Hz, 1H, Bn-H-5), 7.32 (dd, *J* = 8.0, 1.6 Hz, 1H, Bn-H-3), 7.56 (d, *J* = 1.6 Hz, 1H, Bn-H-6), 7.65 (d, *J_trans_* = 16.0 Hz, 1H, H-α); ^13^C-NMR (DMSO-*d*_6_): δ 200.06, 174.38, 173.98, 169.39, 166.87, 151.36, 149.95, 145.88, 128.42, 123.05, 121.13, 118.30, 113.51, 109.11, 81.49, 61.60, 60.50, 55.68, 54.97, 53.49, 48.04, 45.32, 44.37, 43.14, 41.28, 38.08, 38.04, 37.39, 36.84, 32.62, 32.13, 31.80, 31.26, 29.73, 28.49, 28.39, 28.13, 26.46, 26.37, 23.59, 23.29, 18.76, 17.50, 16.76, 16.42, 15.47, 14.27; ESI-MS: *m*/*z* = 854.18 [M + H]^+^. Anal. Calcd. for C_47_H_68_ClNO_8_Se (853.40): C, 63.47; H, 7.71; N, 1.57%. Found: C, 63.44; H, 7.75; N, 1.53%.

*trans-4-Hydroxycinnamic acid methyl ester 3β-(l-methionine)-11-oxo-olean-12-en-30-oate hydrochloride* (**45**)*.* Obtained from **29** as a white solid (143 mg, 76%); m.p. 205–207 °C; ^1^H-NMR (DMSO-*d*_6_): δ 0.71–0.74 (m, 1H, H-5), 0.83 (s, 3H, H-28), 0.84 (s, 3H, H-24), 0.85 (s, 3H, H-23), 0.91–0.93 (m, 1H, H-15’), 1.00–1.04 (m, 1H, H-1’), 1.07 (s, 3H, H-26), 1.08 (s, 3H, H-25), 1.17–1.20 (m, 1H, H-16’), 1.24–1.27 (m, 2H, H-22’ and 21’), 1.33 (s, 3H, H-29), 1.41 (s, 3H, H-27), 1.45–1.48 (m, 1H, H-22), 1.49–1.50 (m, 1H, H-7’), 1.52-1.53 (m, 1H, H-6’), 1.54–1.57 (m, 1H, H-6), 1.64 (dd, *J* = 13.2, 4.0 Hz, 1H, H-19’), 1.68–1.70 (m, 1H, H-2’), 1.71–1.73 (m, 1H, H-7), 1.79–1.82 (m, 1H, H-2), 1.87 (ddd, *J* = 15.2, 15.2, 6.4 Hz, 1H, H-16), 1.93–1.95 (m, 1H, H-21), 1.97–2.00 (m, 1H, H-15), 2.04 (s, 3H, SCH_3_), 2.15–2.17 (m, 1H, H-18), 2.43 (s, 1H, H-9), 2.53–2.55 (m, 1H, H-19), 2.56–2.59 (m, 2H, SCH_2_), 2.64 (ddd, *J* = 12.8, 2.8, 2.8 Hz, 1H, H-1), 3.74 (s, 3H, COOCH_3_), 4.47 (dd, *J* = 10.8, 5.6 Hz, 1H, H-3), 5.46 (s, 1H, H-12), 6.65 (d, *J_trans_* = 16.0 Hz, 1H, H-β), 7.17 (d, *J* = 8.8 Hz. 2H, Bn-H-2 and 6), 7.69 (d, *J* = 8.8 Hz, 2H, Bn-H-3 and 5), 7.81 (d, *J_trans_* = 16.0 Hz, 2H, H-α); ^13^C-NMR (DMSO-*d_6_*): δ 199.80, 174.83, 174.01, 168.76, 166.98, 162.27, 145.45, 132.17, 129.13, 128.58, 121.98, 118.45, 81.48, 61.73, 54.94, 53.68, 51.83, 48.43, 45.36, 44.39, 43.24, 41.10, 38.16, 38.12, 37.78, 37.15, 32.66, 32.43, 31.88, 31.13, 29.98, 28.54, 28.18, 28.14, 26.57, 26.40, 23.63, 23.39, 18.65, 17.33, 16.74, 16.42, 15.54; ESI-MS: *m*/*z* = 784.23 [M + Na]^+^. Anal. Calcd. for C_45_H_64_ClNO_7_S (761.43): C, 67.69; H, 8.08; N, 1.75; S, 4.02%. Found: C, 67.65; H, 8.10; N, 1.69; S, 4.10%.

*trans-4-Hydroxycinnamic acid methyl ester 3β-(l-selenomethionine)-11-oxo-olean-12-en-30-oate hydrochloride* (**46**). Obtained from **30** as a white solid (156 mg, 80%); m.p. 218–221 °C; ^1^H-NMR (DMSO-*d*_6_): δ 0.71–0.74 (m, 1H, H-5), 0.83 (s, 3H, H-28), 0.84 (s, 3H, H-24), 0.85 (s, 3H, H-23), 0.90–0.93 (m, 1H, H-15’), 1.00–1.03 (m, 1H, H-1’), 1.06 (s, 3H, H-26), 1.07 (s, 3H, H-25), 1.16–1.20 (m, 1H, H-16’), 1.23–1.27 (m, 2H, H-22’ and 21’), 1.33 (s, 3H, H-29), 1.41 (s, 3H, H-27), 1.44–1.46 (m, 1H, H-22), 1.48–1.50 (m, 1H, H-7’), 1.52–1.55 (m, 1H, H-6’), 1.56–1.59 (m, 1H, H-6), 1.65 (dd, *J* = 13.2, 4.0 Hz, 1H, H-19’), 1.68–1.69 (m, 1H, H-2’), 1.71–1.74 (m, 1H, H-7), 1.78–1.81 (m, 1H, H-2), 1.86 (ddd, *J* = 15.2, 15.2, 6.4 Hz, 1H, H-16), 1.88–1.90 (m, 1H, H-21), 1.91–1.92 (m, 1H, H-15), 1.93 (s, 3H, SeCH_3_), 2.15–2.18 (m, 1H, H-18), 2.43 (s, 1H, H-9), 2.53–2.55 (m, 1H, H-19), 2.58–2.61 (m, 2H, SeCH_2_), 2.64 (ddd, *J* = 12.8, 2.8, 2.8 Hz, 1H, H-1), 3.73 (s, 3H, COOCH_3_), 4.47 (dd, *J* = 10.8, 5.6 Hz, 1H, H-3), 5.45 (s, 1H, H-12), 6.64 (d, *J_trans_* = 16.0 Hz, 1H, H-β), 7.17 (d, *J* = 8.8 Hz, 2H, Bn-H-2 and 6), 7.69 (d, *J* = 8.8 Hz, 2H, Bn-H-3 and 5), 7.81 (d, *J_trans_* = 16.0 Hz, 2H, H-α); ^13^C-NMR (DMSO-*d*_6_): δ 199.83, 174.83, 173.95, 168.67, 167.03, 162.25, 145.44, 132.20, 129.13, 128.58, 121.97, 118.48, 81.52, 61.73, 54.96, 53.65, 51.83, 48.44, 45.36, 44.35, 43.27, 41.13, 38.18, 38.14, 37.80, 37.15, 32.62, 32.40, 31.93, 31.15, 30.00, 28.59, 28.20, 28.18, 26.59, 26.42, 23.65, 23.40, 18.70, 17.29, 16.72, 16.48, 15.53; ESI-MS: *m*/*z* = 810.14 [M + H]^+^. Anal. Calcd. for C_45_H_64_ClNO_7_Se (809.38): C, 63.93; H, 7.63; N, 1.66%. Found: C, 63.86; H, 7.68; N, 1.61%.

*trans-4-Hydroxycinnamic acid ethyl ester 3β-(l-methionine)-11-oxo-olean-12-en-30-oatehydrochloride* (**47**). Obtained from **31** as a white solid (138 mg, 74%); m.p. 202–204 °C; ^1^H-NMR (DMSO-*d*_6_): δ 0.71–0.74 (m, 1H, H-5), 0.83 (s, 3H, H-28), 0.84 (s, 3H, H-24), 0.85 (s, 3H, H-23), 0.91–0.94 (m, 1H, H-15’), 1.00–1.04 (m, 1H, H-1’), 1.07 (s, 3H, H-26), 1.08 (s, 3H, H-25), 1.13–1.14 (m, 1H, H-16’), 1.18–1.22 (m, 2H, H-22’ and 21’), 1.27 (t, *J* = 7.2 Hz, 3H, CH_3_), 1.33 (s, 3H, H-29), 1.41 (s, 3H, H-27), 1.45–1.47 (m, 1H, H-22), 1.49–1.51 (m, 1H, H-7’), 1.52–1.55 (m, 1H, H-6’), 1.57–1.60 (m, 1H, H-6), 1.64 (dd, *J* = 13.2, 4.0 Hz, 1H, H-19’), 1.67–1.68 (m, 1H, H-2’), 1.71–1.74 (m, 1H, H-7), 1.79–1.82 (m, 1H, H-2), 1.87 (ddd, *J* = 14.8, 14.8, 6.0 Hz, 1H, H-16), 1.93–1.95 (m, 1H, H-21), 1.97–1.99 (m, 1H, H-15), 2.04 (s, 3H, SCH_3_), 2.15–2.17 (m, 1H, H-18), 2.43 (s, 1H, H-9), 2.52–2.54 (m, 1H, H-19), 2.56–2.58 (m, 2H, SCH_2_), 2.64 (ddd, *J* = 13.2, 3.2, 3.2 Hz, 1H, H-1), 4.20 (q, *J* = 7.2 Hz, 2H, CH_2_CH_3_), 4.47 (dd, *J* = 11.2, 5.2 Hz, 1H, H-3), 5.46 (s, 1H, H-12), 6.63 (d, *J_trans_* = 16.0 Hz, 1H, H-β), 7.17 (d, *J* = 8.8 Hz, 2H, Bn-H-2 and 6), 7.67 (d, *J* = 8.8 Hz, 2H, Bn-H-3 and 5), 7.81 (d, *J_trans_* = 16.0 Hz, 2H, H-α); ^13^C-NMR (DMSO-*d*_6_): δ 199.94, 174.85, 174.05, 168.92, 166.91, 162.23, 145.47, 132.17, 129.36, 128.58, 122.02, 118.40, 81.40, 61.62, 60.63, 54.94, 53.68, 48.44, 45.47, 44.40, 43.21, 41.06, 38.05, 38.01, 37.63, 36.90, 32.60, 32.36, 31.88, 31.06, 29.96, 28.52, 28.30, 28.25, 26.46, 26.43, 23.60, 23.36, 18.74, 17.40, 16.75, 16.43, 15.54, 14.46; ESI-MS: *m*/*z* = 776.28 [M + H]^+^. Anal. Calcd. for C_46_H_66_ClNO_7_S (775.45): C, 68.00; H, 8.19; N, 1.72; S, 3.95%. Found: C, 67.96; H, 8.21; N, 1.70; S, 3.97%.

*trans-4-Hydroxycinnamic acid ethyl ester 3β-(l-selenomethionine)-11-oxo-olean-12-en-30-oate hydrochloride* (**48**). Obtained from **32** as a white solid (145 mg, 73%); m.p. 208–211 °C; ^1^H-NMR (DMSO-*d*_6_): δ 0.71–0.74 (m, 1H, H-5), 0.83 (s, 3H, H-28), 0.84 (s, 3H, H-24), 0.85 (s, 3H, H-23), 0.91–0.94 (m, 1H, H-15’), 1.00–1.04 (m, 1H, H-1’), 1.07 (s, 3H, H-26), 1.08 (s, 3H, H-25), 1.13–1.14 (m, 1H, H-16’), 1.18–1.22 (m, 2H, H-22’ and 21’), 1.27 (t, *J* = 7.2 Hz, 3H, CH_3_), 1.33 (s, 3H, H-29), 1.41 (s, 3H, H-27), 1.45–1.47 (m, 1H, H-22), 1.49–1.51 (m, 1H, H-7’), 1.52–1.55 (m, 1H, H-6’), 1.57–1.59 (m, 1H, H-6), 1.65 (dd, *J* = 13.2, 4.0 Hz, 1H, H-19’), 1.68–1.70 (m, 1H, H-2’), 1.71–1.73 (m, 1H, H-7), 1.78–1.80 (m, 1H, H-2), 1.87 (ddd, *J* = 14.8, 14.8, 6.0 Hz, 1H, H-16), 1.89–1.91 (m, 1H, H-21), 1.92–1.93 (m, 1H, H-15), 1.94 (s, 3H, SeCH_3_), 2.15–2.18 (m, 1H, H-18), 2.43 (s, 1H, H-9), 2.52–2.54 (m, 1H, H-19), 2.56–2.59 (m, 2H, SeCH_2_), 2.64 (ddd, *J* = 13.2, 3.2, 3.2 Hz, 1H, H-1), 4.20 (q, *J* = 7.2 Hz, 2H, CH_2_CH_3_), 4.47 (dd, *J* = 11.2, 5.2 Hz, 1H, H-3), 5.46 (s, 1H, H-12), 6.63 (d, *J_trans_* = 16.0 Hz, 1H, H-β), 7.17 (d, *J* = 8.8 Hz, 2H, Bn-H-2 and 6), 7.67 (d, *J* = 8.8 Hz, 2H, Bn-H-3 and 5), 7.81 (d, *J_trans_* = 16.0 Hz, 2H, H-α); ^13^C-NMR (DMSO-*d*_6_): δ 199.92, 174.88, 173.98, 168.98, 166.88, 162.25, 145.49, 132.19, 129.28, 128.56, 122.06, 118.45, 81.49, 61.62, 60.59, 54.96, 53.64, 48.49, 45.43, 44.36, 43.26, 41.03, 38.05, 38.04, 37.65, 36.89, 32.60, 32.33, 31.88, 31.10, 29.97, 28.59, 28.32, 28.26, 26.44, 26.37, 23.59, 23.35, 18.75, 17.38, 16.77, 16.46, 15.54, 14.48; ESI-MS: *m*/*z* = 846.32 [M + Na]^+^. Anal. Calcd. for C_46_H_66_ClNO_7_Se (823.39): C, 64.29; H, 7.74; N, 1.63%. Found: C, 64.25; H, 7.77; N, 1.59%.

*Isoferulic acid methyl ester 3β-(l-methionine)-11-oxo-olean-12-en-30-oate hydrochloride* (**49**)*.* Obtained from **33** as a white solid (143 mg, 75%); m.p. 199–201 °C; ^1^H-NMR (DMSO-*d*_6_): δ 0.71–0.74 (m, 1H, H-5), 0.84 (s, 3H, H-28), 0.85 (s, 3H, H-24), 0.85 (s, 3H, H-23), 0.91–0.94 (m, 1H, H-15’), 1.00–1.03 (m, 1H, H-1’), 1.08 (s, 3H, H-26), 1.08 (s, 3H, H-25), 1.12–1.14 (m, 1H, H-16’), 1.20–1.24 (m, 2H, H-22’ and 21’), 1.33 (s, 3H, H-29), 1.41 (s, 3H, H-27), 1.46 (m, 1H, H-22), 1.50 (m, 1H, H-7’), 1.52 (m, 1H, H-6’), 1.55 (m, 1H, H-6), 1.64 (dd, 1H, *J* = 13.2, 4.0 Hz, H-19’), 1.68 (m, 1H, H-2’), 1.71 (m, 1H, H-7), 1.80 (m, 1H, H-2), 1.87 (ddd, 1H, *J* = 14.8, 14.8, 5.6 Hz, H-16), 1.92 (m, 1H, H-21), 1.95 (m, 1H, H-15), 2.04 (s, 3H, SCH_3_), 2.28 (m, 1H, H-18), 2.43 (s, 1H, H-9), 2.54 (m, 1H, H-19), 2.57 (m, 2H, SCH_2_), 2.64 (ddd, 1H, *J* = 12.8, 3.2, 3.2 Hz, H-1), 3.72 (s, 3H, COOCH_3_), 3.82 (s, 3H, OCH_3_), 4.47 (dd, 1H, *J* = 11.2, 5.2 Hz, H-3), 5.48 (s, 1H, H-12), 6.60 (d, 1H, *J_trans_* = 16.0 Hz, H-β), 7.18 (d, 1H, *J* = 8.4 Hz, Bn-H-3), 7.57 (d, 1H, *J* = 2.0 Hz, Bn-H-6), 7.62 (dd, 1H, *J* = 8.4, 2.0 Hz, Bn-H-4), 7.63 (d, 1H, *J_trans_* = 16.0 Hz, H-α); ^13^C-NMR (DMSO-*d*_6_): δ 200.03, 174.45, 172.81, 169.03, 166.97, 152.83, 143.64, 138.11, 128.42, 127.43, 127.38, 121.70, 116.21, 112.29, 81.49, 61.60, 55.71, 54.89, 52.80, 51.55, 47.91, 45.32, 44.39, 43.03, 41.13, 38.95, 38.89, 37.84, 36.90, 32.63, 32.15, 31.84, 31.21, 29.63, 28.51, 28.35, 28.12, 26.35, 26.29, 23.59, 23.15, 18.67, 17.40, 16.73, 16.27, 15.41; ESI-MS: *m*/*z* = 814.21 [M + Na]^+^. Anal. Calcd. for C_46_H_66_ClNO_8_S (791.44): C, 66.68; H, 8.03; N, 1.69; S, 3.87%. Found: C, 66.62; H, 8.08; N, 1.66; S, 3.91%.

*Isoferulic acid methyl ester 3β-(l-selenomethionine)-11-oxo-olean-12-en-30-oate hydrochloride* (**50**)*.* Obtained from **34** as a white solid (158 mg, 78%); m.p. 201–203 °C; ^1^H-NMR (DMSO-*d*_6_): δ 0.71–0.74 (m, 1H, H-5), 0.84 (s, 3H, H-28), 0.85 (s, 3H, H-24), 0.85 (s, 3H, H-23), 0.91–0.94 (m, 1H, H-15’), 1.00–1.03 (m, 1H, H-1’), 1.08 (s, 3H, H-26), 1.08 (s, 3H, H-25), 1.13–1.15 (m, 1H, H-16’), 1.20–1.24 (m, 2H, H-22’ and 21’), 1.33 (s, 3H, H-29), 1.41 (s, 3H, H-27), 1.46 (m, 1H, H-22), 1.50 (m, 1H, H-7’), 1.52 (m, 1H, H-6’), 1.55 (m, 1H, H-6), 1.64 (dd, 1H, *J* = 13.2, 4.0 Hz, H-19’), 1.68 (m, 1H, H-2’), 1.71 (m, 1H, H-7), 1.80 (m, 1H, H-2), 1.86 (ddd, 1H, *J* = 14.8, 14.8, 5.6 Hz, H-16), 1.89 (m, 1H, H-21), 1.92 (m, 1H, H-15), 1.93 (s, 3H, SeCH_3_), 2.28 (m, 1H, H-18), 2.43 (s, 1H, H-9), 2.55 (m, 1H, H-19), 2.58 (m, 2H, SeCH_2_), 2.64 (ddd, 1H, *J* = 12.8, 3.2, 3.2 Hz, H-1), 3.72 (s, 3H, COOCH_3_), 3.82 (s, 3H, OCH_3_), 4.47 (dd, 1H, *J* = 11.2, 5.2 Hz, H-3), 5.48 (s, 1H, H-12), 6.60 (d, 1H, *J_trans_* = 16.0 Hz, H-β), 7.18 (d, 1H, *J* = 8.4 Hz, Bn-H-3), 7.57 (d, 1H, *J* = 2.0 Hz, Bn-H-6), 7.62 (dd, 1H, *J* = 8.4, 2.0 Hz, Bn-H-4), 7.63 (d, 1H, *J_trans_* = 16.0 Hz, H-α); ^13^C-NMR (DMSO-*d*_6_): δ 200.05, 174.43, 172.75, 169.11, 166.97, 152.85, 143.64, 138.13, 128.43, 127.43, 127.38, 121.70, 116.21, 112.26, 81.42, 61.56, 55.76, 54.91, 52.76, 51.53, 47.96, 45.31, 44.45, 43.06, 41.14, 39.00, 38.85, 37.86, 36.91, 32.65, 32.15, 31.85, 31.26, 29.66, 28.52, 28.33, 28.13, 26.38, 26.30, 23.54, 23.14, 18.70, 17.33, 16.74, 16.30, 15.40; ESI-MS: *m*/*z* = 840.28 [M + H]^+^. Anal. Calcd. for C_46_H_66_ClNO_8_Se (839.39): C, 63.11; H, 7.60; N, 1.60%. Found: C, 63.05; H, 7.64; N, 1.53%.

*Isoferulic acid ethyl ester 3β-(l-methionine)-11-oxo-olean-12-en-30-oate hydrochloride* (**51**)*.* Obtained from **35** as a white solid (144 mg, 74%); m.p. 192–195 °C; ^1^H-NMR (DMSO-*d*_6_): δ 0.71–0.74 (m, 1H, H-5), 0.84 (s, 3H, H-28), 0.85 (s, 3H, H-24), 0.85 (s, 3H, H-23), 0.91–0.94 (m, 1H, H-15’), 1.00–1.04 (m, 1H, H-1’), 1.08 (s, 3H, H-26), 1.08 (s, 3H, H-25), 1.13–1.15 (m, 1H, H-16’), 1.18–1.21 (m, 2H, H-22’ and 21’), 1.26 (t, 3H, *J* = 7.2 Hz, CH_3_), 1.33 (s, 3H, H-29), 1.41 (s, 3H, H-27), 1.46 (m, 1H, H-22), 1.50 (m, 1H, H-7’), 1.52 (m, 1H, H-6’), 1.54 (m, 1H, H-6), 1.64 (dd, 1H, *J* = 13.2, 4.0 Hz, H-19’), 1.68 (m, 1H, H-2’), 1.71 (m, 1H, H-7), 1.80 (m, 1H, H-2), 1.86 (ddd, 1H, *J* = 14.4, 14.4, 5.6 Hz, H-16), 1.89 (m, 1H, H-21), 1.92 (m, 1H, H-15), 2.04 (s, 3H, SCH_3_), 2.29 (m, 1H, H-18), 2.43 (s, 1H, H-9), 2.53 (m, 1H, H-19), 2.56 (m, 2H, SCH_2_), 2.64 (ddd, 1H, *J* = 13.6, 3.6, 3.6 Hz, H-1), 3.82 (s, 3H, OCH_3_), 4.18 (q, 2H, *J* = 7.2 Hz, CH_2_CH_3_), 4.47 (dd, 1H, *J* = 11.6, 4.8 Hz, H-3), 5.48 (s, 1H, H-12), 6.59 (d, 1H, *J_trans_* = 16.0 Hz, H-β), 7.18 (d, 1H, *J* = 8.4 Hz, Bn-H-3), 7.59 (d, 1H, *J* = 2.0 Hz, Bn-H-6), 7.61 (dd, 1H, *J* = 8.4, 2.0 Hz, Bn-H-4), 7.62 (d, 1H, *J_trans_* = 16.0 Hz, H-α); ^13^C-NMR (DMSO-*d*_6_): δ 200.21, 174.46, 172.85, 169.25, 166.90, 152.80, 143.61, 138.08, 128.40, 127.41, 127.38, 121.65, 116.17, 112.26, 81.35, 61.65, 60.65, 55.70, 54.87, 53.47, 47.96, 45.27, 44.27, 43.08, 41.17, 38.99, 38.85, 37.80, 36.87, 32.61, 32.18, 31.80, 31.20, 29.70, 28.47, 28.42, 28.17, 26.37, 26.30, 23.64, 23.13, 18.72, 17.38, 16.70, 16.34, 15.43, 14.23; ESI-MS: *m*/*z* = 828.33 [M + Na]^+^. Anal. Calcd. for C_47_H_68_ClNO_8_S (805.46): C, 67.00; H, 8.13; N, 1.66; S, 3.81%. Found: C, 66.96; H, 8.15; N, 1.61; S, 3.84%.

*Isoferulic acid ethyl ester 3β-(l-selenomethionine)-11-oxo-olean-12-en-30-oate hydrochloride* (**52**)*.* Obtained from **36** as a white solid (154 mg, 75%); m.p. 196–198 °C; ^1^H-NMR (DMSO-*d*_6_): δ 0.71–0.75 (m, 1H, H-5), 0.84 (s, 3H, H-28), 0.85 (s, 3H, H-24), 0.85 (s, 3H, H-23), 0.91–0.93 (m, 1H, H-15’), 1.00–1.04 (m, 1H, H-1’), 1.08 (s, 3H, H-26), 1.08 (s, 3H, H-25), 1.13–1.15 (m, 1H, H-16’), 1.18–1.21 (m, 2H, H-22’ and 21’), 1.26 (t, 3H, *J* = 7.2 Hz, CH_3_), 1.33 (s, 3H, H-29), 1.41 (s, 3H, H-27), 1.46 (m, 1H, H-22), 1.50 (m, 1H, H-7’), 1.52 (m, 1H, H-6’), 1.54 (m, 1H, H-6), 1.64 (dd, 1H, *J* = 13.2, 4.0 Hz, H-19’), 1.68 (m, 1H, H-2’), 1.70 (m, 1H, H-7), 1.80 (m, 1H, H-2), 1.86 (ddd, 1H, *J* = 14.4, 14.4, 5.6 Hz, H-16), 1.89 (m, 1H, H-21), 1.92 (m, 1H, H-15), 1.94 (s, 3H, SeCH_3_), 2.30 (m, 1H, H-18), 2.43 (s, 1H, H-9), 2.55 (m, 1H, H-19), 2.58 (m, 2H, SeCH_2_), 2.64 (ddd, 1H, *J* = 13.6, 3.6, 3.6 Hz, H-1), 3.82 (s, 3H, OCH_3_), 4.18 (q, 2H, *J* = 7.2 Hz, CH_2_CH_3_), 4.47 (dd, 1H, *J* = 11.6, 4.8 Hz, H-3), 5.48 (s, 1H, H-12), 6.59 (d, 1H, *J_trans_* = 16.0 Hz, H-β), 7.18 (d, 1H, *J* = 8.4 Hz, Bn-H-3), 7.59 (d, 1H, *J* = 2.0 Hz, Bn-H-6), 7.61 (dd, 1H, *J* = 8.4, 2.0 Hz, Bn-H-4), 7.62 (d, 1H, *J_trans_* = 16.0 Hz, H-α); ^13^C-NMR (DMSO-*d*_6_): δ 200.24, 174.41, 172.87, 169.20, 166.96, 152.83, 143.62, 138.08, 128.43, 127.41, 127.36, 121.65, 116.20, 112.25, 81.33, 61.65, 60.65, 55.72, 54.91, 53.45, 47.94, 45.27, 44.27, 43.06, 41.18, 38.96, 38.86, 37.82, 36.88, 32.61, 32.15, 31.83, 31.23, 29.68, 28.48, 28.45, 28.15, 26.39, 26.30, 23.60, 23.15, 18.74, 17.42, 16.75, 16.37, 15.47, 14.25; ESI-MS: *m*/*z* = 854.24 [M + H]^+^. Anal. Calcd. for C_47_H_68_ClNO_8_Se (853.40): C, 63.47; H, 7.71; N, 1.57%. Found: C, 63.44; H, 7.74; N, 1.52%.

*trans-3-Hydroxycinnamic acid methyl ester 3β-(l-methionine)-11-oxo-olean-12-en-30-oate hydrochloride* (**53**)*.* Obtained from **37** as a white solid (145 mg, 79%); m.p. 190–192 °C; ^1^H-NMR (DMSO-*d*_6_): δ 0.72–0.74 (m, 1H, H-5), 0.84 (s, 3H, H-28), 0.85 (s, 3H, H-24), 0.85 (s, 3H, H-23), 0.91–0.93 (m, 1H, H-15’), 1.00–1.04 (m, 1H, H-1’), 1.06 (s, 3H, H-26), 1.07 (s, 3H, H-25), 1.12–1.14 (m, 1H, H-16’), 1.17–1.20 (m, 2H, H-22’ and 21’), 1.34 (s, 3H, H-29), 1.41 (s, 3H, H-27), 1.45 (m, 1H, H-22), 1.49 (m, 1H, H-7’), 1.52 (m, 1H, H-6’), 1.54 (m, 1H, H-6), 1.65 (dd, 1H, *J* = 13.2, 4.0 Hz, H-19’), 1.68 (m, 1H, H-2’), 1.70 (m, 1H, H-7), 1.79 (m, 1H, H-2), 1.86 (ddd, 1H, *J* = 15.2, 15.2, 6.4 Hz, H-16), 1.94 (m, 1H, H-21), 1.97 (m, 1H, H-15), 2.03 (s, 3H, SCH_3_), 2.18 (m, 1H, H-18), 2.43 (s, 1H, H-9), 2.52 (m, 1H, H-19), 2.54 (m, 2H, SCH_2_), 2.63 (ddd, 1H, *J* = 12.8, 2.8, 2.8 Hz, H-1), 3.74 (s, 3H, COOCH_3_), 4.47 (dd, 1H, *J* = 10.8, 5.6 Hz, H-3), 5.48 (s, 1H, H-12), 6.74 (d, 1H, *J_trans_* = 16.0 Hz, H-β), 7.15 (dd, 1H, *J* = 8.0, 2.0 Hz, Bn-H-6), 7.48 (t, 1H, *J* = 8.0 Hz, Bn-H-5), 7.54 (s, 1H, Bn-H-2), 7.65 (d, 1H, *J* = 7.6 Hz, Bn-H-4), 7.71 (d, 1H, *J_trans_* = 16.0 Hz, H-α); ^13^C-NMR (DMSO-*d*_6_): δ 199.80, 174.87, 173.94, 168.82, 167.07, 151.16, 143.69, 135.91, 129.83, 128.57, 125.59, 123.26, 120.65, 118.94, 81.50, 61.61, 54.92, 53.63, 51.76, 48.36, 45.32, 44.28, 43.15, 40.94, 38.57, 38.07, 37.72, 36.84, 32.59, 32.41, 31.88, 31.07, 29.95, 28.57, 28.26, 28.16, 26.49, 26.32, 23.61, 23.33, 18.61, 17.28, 16.75, 16.31, 15.54; ESI-MS: *m*/*z* = 762.27 [M + H]^+^. Anal. Calcd. for C_45_H_64_ClNO_7_S (761.43): C, 67.69; H, 8.08; N, 1.75; S, 4.02%. Found: C, 67.64; H, 8.13; N, 1.72; S, 4.10%.

*trans-3-Hydroxycinnamic acid methyl ester 3β-(l-selenomethionine)-11-oxo-olean-12-en-30-oate hydro-chloride* (**54**)*.* Obtained from **38** as a white solid (148 mg, 76%); m.p. 194–196 °C; ^1^H-NMR (DMSO-*d*_6_): δ 0.72–0.74 (m, 1H, H-5), 0.84 (s, 3H, H-28), 0.85 (s, 3H, H-24), 0.85 (s, 3H, H-23), 0.90–0.93 (m, 1H, H-15’), 1.00–1.03 (m, 1H, H-1’), 1.06 (s, 3H, H-26), 1.07 (s, 3H, H-25), 1.12–1.14 (m, 1H, H-16’), 1.17–1.20 (m, 2H, H-22’ and 21’), 1.34 (s, 3H, H-29), 1.41 (s, 3H, H-27), 1.44 (m, 1H, H-22), 1.49 (m, 1H, H-7’), 1.52 (m, 1H, H-6’), 1.54 (m, 1H, H-6), 1.65 (dd, 1H, *J* = 13.2, 4.0 Hz, H-19’), 1.68 (m, 1H, H-2’), 1.70 (m, 1H, H-7), 1.75 (m, 1H, H-2), 1.86 (ddd, 1H, *J* = 15.2, 15.2, 6.4 Hz, H-16), 1.89 (m, 1H, H-21), 1.91 (m, 1H, H-15), 1.93 (s, 3H, SeCH_3_), 2.18 (m, 1H, H-18), 2.43 (s, 1H, H-9), 2.55 (m, 1H, H-19), 2.60 (m, 2H, SeCH_2_), 2.63 (ddd, 1H, *J* = 12.8, 2.8, 2.8 Hz, H-1), 3.74 (s, 3H, COOCH_3_), 4.47 (dd, 1H, *J* = 10.8, 5.6 Hz, H-3), 5.49 (s, 1H, H-12), 6.74 (d, 1H, *J_trans_* = 16.0 Hz, H-β), 7.15 (dd, 1H, *J* = 8.0, 2.0 Hz, Bn-H-6), 7.48 (t, 1H, *J* = 8.0 Hz, Bn-H-5), 7.54 (s, 1H, Bn-H-2), 7.65 (d, 1H, *J* = 7.6 Hz, Bn-H-4), 7.71 (d, 1H, *J_trans_* = 16.0 Hz, H-α); ^13^C-NMR (DMSO-*d*_6_): δ 199.82, 174.87, 173.89, 168.78, 167.09, 151.12, 143.63, 136.03, 129.88, 128.55, 125.53, 123.25, 120.67, 118.97, 81.56, 61.61, 54.93, 53.60, 51.76, 48.37, 45.32, 44.26, 43.16, 40.95, 38.59, 38.07, 37.76, 36.81, 32.63, 32.43, 31.88, 31.13, 29.94, 28.58, 28.27, 28.18, 26.45, 26.33, 23.66, 23.34, 18.68, 17.25, 16.79, 16.34, 15.57; ESI-MS: *m*/*z* = 832.18 [M + Na]^+^. Anal. Calcd. for C_45_H_64_ClNO_7_Se (809.38): C, 63.93; H, 7.63; N, 1.66%. Found: C, 63.90; H, 7.66; N, 1.63%.

*trans-3-Hydroxycinnamic acid ethyl ester 3β-(l-methionine)-11-oxo-olean-12-en-30-oate hydrochloride* (**55**)*.* Obtained from **39** as a white solid (136 mg, 73%); m.p. 183–185 °C; ^1^H-NMR (DMSO-*d*_6_): δ 0.72–0.74 (m, 1H, H-5), 0.84 (s, 3H, H-28), 0.85 (s, 3H, H-24), 0.85 (s, 3H, H-23), 0.91–0.93 (m, 1H, H-15’), 1.00–1.04 (m, 1H, H-1’), 1.07 (s, 3H, H-26), 1.08 (s, 3H, H-25), 1.12–1.14 (m, 1H, H-16’), 1.17–1.20 (m, 2H, H-22’ and 21’), 1.27 (t, 3H, *J* = 7.2 Hz, CH3), 1.34 (s, 3H, H-29), 1.42 (s, 3H, H-27), 1.45 (m, 1H, H-22), 1.49 (m, 1H, H-7’), 1.52 (m, 1H, H-6’), 1.55 (m, 1H, H-6), 1.65 (dd, 1H, *J* = 13.2, 4.0 Hz, H-19’), 1.68 (m, 1H, H-2’), 1.70 (m, 1H, H-7), 1.79 (m, 1H, H-2), 1.86 (ddd, 1H, *J* = 14.8, 14.8, 6.0 Hz, H-16), 1.95 (m, 1H, H-21), 1.98 (m, 1H, H-15), 2.04 (s, 3H, SCH_3_), 2.19 (m, 1H, H-18), 2.43 (s, 1H, H-9), 2.52 (m, 1H, H-19), 2.56 (m, 2H, SCH_2_), 2.64 (ddd, 1H, *J* = 13.2, 3.2, 3.2 Hz, H-1), 4.20 (q, 2H, *J* = 7.2 Hz, CH_2_CH_3_), 4.48 (dd, 1H, *J* = 11.2, 5.2 Hz, H-3), 5.49 (s, 1H, H-12), 6.74 (d, 1H, *J_trans_* = 16.0 Hz, H-β), 7.15 (dd, 1H, *J* = 8.0, 2.0 Hz, Bn-H-6), 7.48 (t, 1H, *J* = 8.0 Hz, Bn-H-5), 7.56 (s, 1H, Bn-H-2), 7.64 (d, 1H, *J* = 7.6 Hz, Bn-H-4), 7.70 (d, 1H, *J_rans_* = 16.0 Hz, H-α); ^13^C-NMR (DMSO-*d*_6_): δ 200.03, 174.81, 172.05, 169.08, 167.02, 151.03, 143.58, 135.87, 129.79, 128.49, 125.58, 123.17, 120.60, 118.79, 81.48, 61.73, 60.61, 54.92, 53.65, 48.36, 45.43, 44.21, 43.16, 40.97, 38.58, 38.12, 37.66, 36.87, 32.55, 32.36, 31.85, 31.17, 29.98, 28.57, 28.35, 28.19, 26.41, 26.42, 23.79, 23.33, 18.76, 17.37, 16.75, 16.48, 15.58, 14.51; ESI-MS: *m*/*z* = 776.34 [M + H]^+^. Anal. Calcd. for C_46_H_66_ClNO_7_S (775.45): C, 68.00; H, 8.19; N, 1.72; S, 3.95%. Found: C, 67.97; H, 8.22; N, 1.68; S, 4.01%.

*trans-3-Hydroxycinnamic acid ethyl ester 3β-(l-selenomethionine)-11-oxo-olean-12-en-30-oate hydrochloride* (**56**)*.* Obtained from **40** as a white solid (149 mg, 75%); m.p. 191–194 °C; ^1^H-NMR (DMSO-*d*_6_): δ 0.72–0.75 (m, 1H, H-5), 0.84 (s, 3H, H-28), 0.85 (s, 3H, H-24), 0.85 (s, 3H, H-23), 0.91–0.93 (m, 1H, H-15’), 1.01–1.04 (m, 1H, H-1’), 1.07 (s, 3H, H-26), 1.08 (s, 3H, H-25), 1.13–1.15 (m, 1H, H-16’), 1.17–1.21 (m, 2H, H-22’ and 21’), 1.27 (t, 3H, *J* = 7.2 Hz, CH_3_), 1.35 (s, 3H, H-29), 1.42 (s, 3H, H-27), 1.45 (m, 1H, H-22), 1.48 (m, 1H, H-7’), 1.52 (m, 1H, H-6’), 1.57 (m, 1H, H-6), 1.65 (dd, 1H, *J* = 13.2, 4.0 Hz, H-19’), 1.68 (m, 1H, H-2’), 1.71 (m, 1H, H-7), 1.76 (m, 1H, H-2), 1.86 (ddd, 1H, *J* = 14.8, 14.8, 6.0 Hz, H-16), 1.89 (m, 1H, H-21), 1.91 (m, 1H, H-15), 1.94 (s, 3H, SeCH_3_), 2.19 (m, 1H, H-18), 2.44 (s, 1H, H-9), 2.54 (m, 1H, H-19), 2.59 (m, 2H, SeCH_2_), 2.64 (ddd, 1H, *J* = 13.2, 3.2, 3.2 Hz, H-1), 4.20 (q, 2H, *J* = 7.2 Hz, CH_2_CH_3_), 4.48 (dd, 1H, *J* = 11.2, 5.2 Hz, H-3), 5.49 (s, 1H, H-12), 6.74 (d, 1H, *J_trans_* = 16.0 Hz, H-β), 7.15 (dd, 1H, *J* = 8.0, 2.0 Hz, Bn-H-6), 7.48 (t, 1H, *J* = 8.0 Hz, Bn-H-5), 7.56 (s, 1H, Bn-H-2), 7.65 (d, 1H, *J* = 8.0 Hz), 7.69 (d, 1H, *J_trans_* = 16.0 Hz, H-α); ^13^C-NMR (DMSO-*d*_6_): δ 200.02, 174.82, 171.96, 169.03, 167.05, 151.08, 143.60, 135.91, 129.72, 128.54, 125.49, 123.13, 120.62, 118.78, 81.44, 61.75, 60.58, 54.95, 53.62, 48.38, 45.38, 44.23, 43.19, 40.98, 38.57, 38.09, 37.65, 36.89, 32.58, 32.33, 31.84, 31.13, 29.96, 28.63, 28.40, 28.13, 26.46, 26.47, 23.65, 23.30, 18.72, 17.39, 16.78, 16.45, 15.60, 14.53; ESI-MS: *m*/*z* = 824.24 [M + H]^+^. Anal. Calcd. for C_46_H_66_ClNO_7_Se (823.39): C, 64.29; H, 7.74; N, 1.63%. Found: C, 64.25; H, 7.77; N, 1.58%.

### 3.7. Anticancer Assays

The cytotoxicities of the compounds were evaluated by the MTT assay, which is based on the conversion of MTT to formazan crystals by mitochondrial dehydrogenases. The three lines of cells (MCF-7, MDA-MB-231 and hTERT-RPE1) were cultured in 96 well plates for 24 h with a density of 0.8 × 10^4^ cells/well, afterwards treated them with varying concentrations of GA, doxorubicin or the derivatives for 24 h at 37 °C. The medium was incubated with 20 μL of 5 mg/mL MTT solution for 3 h in a humidified incubator containing 5% CO_2_. The purple coloured formazan crystals formed in the wells were dissolved in DMSO and their absorbances were measured at 570 nm with a microplate reader. Cell viability was expressed as a percentage of the value in control cultures.

## 4. Conclusions

In summary, forty novel glycyrrhetinic acid derivates were designed, synthesized and evaluated for their inhibitory activities as novel anticancer agents against two human breast cancer cell lines (MCF-7 and MDA-MB-231) and one normal human retinal pigment epithelial cell line (hTERT-RPE1). Most of the prepared compounds displayed moderate cytotoxic activity against both cancer cell lines and relatively lower inhibitory activity against the normal cell line. In particular, compound **42** exhibited good antiproliferative activity, with IC_50_ values of 1.88 ± 0.20 and 1.37 ± 0.18 μM. The results demonstrated that compound **42** could be a potential antitumor agent that deserves further research. Our SAR study disclosed that incorporation of a lipophilic fragment or amino acid groups into an anticancer natural product could increase the activity, compared with the parent compound. Further studies in this area are in progress in our laboratory and will be reported in the future.
